# Aquatic food loss and waste rate in the United States is half of earlier estimates

**DOI:** 10.1038/s43016-023-00881-z

**Published:** 2023-12-13

**Authors:** David C. Love, Frank Asche, Jillian Fry, Ly Nguyen, Jessica Gephart, Taryn M. Garlock, Lekelia D. Jenkins, James L. Anderson, Mark Brown, Silvio Viglia, Elizabeth M. Nussbaumer, Roni Neff

**Affiliations:** 1 Johns Hopkins Center for a Livable Future, Johns Hopkins University, Baltimore, MD USA; 2 Department of Environmental Health and Engineering, Bloomberg School of Public Health, Baltimore, MD USA; 3 School of Forest, Fisheries and Geomatics Sciences, University of Florida, Gainesville, FL USA; 4 Department of Safety, Economics and Planning, University of Stavanger, Stavanger, Norway; 5 Department of Health Sciences, College of Health Professions, Towson University, Towson, MD USA; 6 Food and Resource Economics Department, University of Florida, Gainesville, FL USA; 7 Department of Environmental Science, American University, Washington, DC USA; 8 School of Fisheries, Aquaculture, and Aquatic Sciences, Auburn University, Auburn, AL USA; 9 School for the Future of Innovation in Society, Arizona State University, Tempe, AZ USA; 10 Center for Environmental Policy, University of Florida, Gainesville, FL USA; 11 ENEA, Italian National Agency for New Technologies, Energy and Sustainable Economic Development, Casaccia Research Centre, Rome, Italy

**Keywords:** Environmental impact, Agriculture

## Abstract

Food loss and waste (FLW) is a major challenge to food system sustainability, including aquatic foods. We investigated aquatic FLW in the food supply of the United States, the largest importer of aquatic food globally, using primary and secondary data and life cycle methodology. We show that there are significant differences in FLW among species, production technology, origin and stage of supply chain. We estimate total aquatic FLW was 22.7%, which is 43–55% lower than earlier estimates reported in the literature, illustrating the importance of applying a disaggregated approach. Production losses associated with imported food contribute over a quarter of total FLW, and addressing these losses requires multinational efforts to implement interventions along the supply chain. These findings inform prioritization of solutions—including areas of need for innovations, government incentives, policy change, infrastructure and equity.

## Abstract (web summary)

Data on aquatic food loss and waste are limited outside small-scale fisheries, and major gaps exist for aquaculture species that make up half of global production. This study estimates loss and waste in the US aquatic food supply chain from 2014 to 2018, including the top ten species groups that together represent 89% of the country’s aquatic food supply.

## Main

Aquatic foods play an important role in diets around the world^1–4^. There is significant literature on the losses in capture fisheries, but it is largely driven by case studies, and there are important omissions. There is a limited focus on loss among aquaculture species, despite aquaculture now making up about 50% of global edible production^5^, and many studies focus on the ends of the supply chains (that is, the production or consumer side), while food loss in the middle of supply chains is not studied. Here we investigate food loss in the aquatic food supply of the United States, the largest importer of aquatic food globally, as an example of how to conduct waste and loss estimates in complex multi-country supply chains.

Forty-four percent of aquatic foods (70 million tonnes) globally are sold live or fresh^5^ and are highly perishable if not subsequently preserved^6^. The large reliance on live and fresh product forms is partly due to their higher retail value than frozen or shelf-stable forms^7^, but this requires dependable cold chain management. In addition, most aquatic food has a characteristic smell when unrefrigerated^8^, which can cause perceived food safety concerns and potentially result in greater losses compared with other foods.

Reducing food loss is an important factor in improving global food security and planetary health^9–12^. The United Nations (UN) Sustainable Development Goal 12.3 includes halving food loss at retail and consumer levels by 2030 and reducing food loss in production and supply^5^. We use the UN Food and Agriculture Organization (FAO) definition of ‘food loss’ as a decrease in the quantity or quality of food in production and distribution, and ‘food waste’ as the removal of edible food from the food supply by choice, spoilage or food expiration^13^. Food waste is often considered a subset of loss and typically arises at the consumer stage, and we refer collectively to these losses as food loss and waste (FLW). Accurate and reliable data on FLW are urgently needed in many countries, sectors and supply chains to track progress towards policy goals and refine interventions^14,15^.

In 2011, the FAO estimated 35% of aquatic foods are lost and wasted globally, which was higher than cereals (30%), oilseeds (20%), and meat and dairy (20%), but lower than root crops, and fruits and vegetables (40–50%)^1,2^. The FAO estimate showed that 50% of aquatic foods in North America were lost and wasted, which was among the highest rates of FLW for any food group in the world^2^. Although these estimates are more than a decade old, they continue to be used by research, policy and advocacy communities, and we will show that updated estimates reduce the estimates by 43–55%.

To inform strategies for meeting FLW targets, the 2011 FAO estimates for aquatic foods must be updated and improved to address four assumptions used in earlier modelling^2,16^. The first assumption used in the FAO estimate was that aquatic FLW only comes from wild capture fisheries. Today, half of global aquatic food supply comes from aquaculture (that is, farm raised)^5^, making aquaculture an important component of diets and probably also an important source of FLW. The second assumption is that loss only arises from regional production and did not consider trade; however, aquatic foods are the most traded major food group^5,17^. The third assumption is that all aquatic foods were only sold at retail outlets. Recent work has shown that food service represents a significant share (39% in the United States) of aquatic food sales by volume^18^. Last, consumer waste estimates were based on indirect methods (that is, national retail sales compared to national consumption)^19^, which is not as accurate as direct measurements of household-level FLW. In 2019, the FAO replaced their 2011 study, citing many of the same limitations listed above^20^; however, the replacement report combines aquatic foods with meat and animal products, and the underlying database only has a single entry for aquatic foods (snails)^21^.

Literature on aquatic FLW is largely driven by case studies in the small-scale capture fisheries sector and their supply chains in low- and middle-income countries (LMICs)^22^. This leaves large gaps in understanding of losses among large-scale capture fisheries, large- and small-scale aquaculture species, and supply chains for high-income countries. FLW can vary by species and origin; uses and yields vary significantly as aquatic foods are produced and even re-processed in various supply chains^23^. Additionally, most US food waste research lumps aquatic foods with meat and poultry in reporting^24–27^, although separate data are sometimes collected^28^. While important similarities exist between aquatic foods and terrestrial meats, such as perishability and position on consumer plates, aquatic food is distinguished by factors including its variety of production methods and species, import patterns, distance of fishing sites from land, reliance on water quality, fragility, consumer familiarity and odours. Each of these factors shapes FLW patterns and opportunities for responses.

**Fig. 1:**
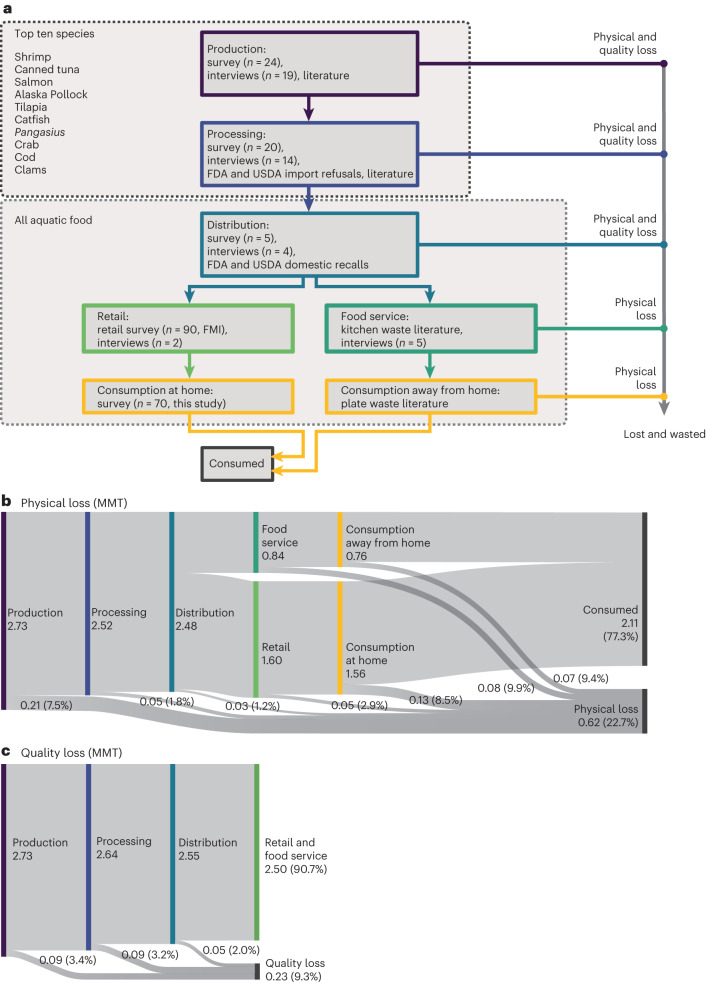
2014–2018 average human-edible aquatic FLW in the United States. **a**, Study flowchart and data sources. FDA, US Food and Drug Administration; FMI, Food Marketing Institute; USDA, US Department of Agriculture. **b**,**c**, Sankey diagram showing loss (MMT, %) within each stage for physical loss (**b**) and quality loss (**c**). Quality loss was not available for the food service, retail and consumer stages. Width of the Sankey bands are proportional to the amount of product consumed or lost. Values are reported in Supplementary Data 1.

Here we aim to improve on the FAO estimate for aquatic FLW in the United States using an extensive primary data collection effort across the top ten fishery and aquaculture supply chains serving the United States, and supplemented by secondary data and literature to enable generation of national estimates. The study boundary begins at production (that is, farm or fishery), including production outside the United States, and ends when aquatic foods were consumed in the United States or removed from the supply chain. Overall loss and waste estimates are provided for the US supply, one of the world’s largest fishing nations and importers of aquatic foods^29^, for all stages of the supply chain from farm or fishery to plate from 2014 to 2018 (Fig. 1a).

## Results

### Overall aquatic FLW

The US edible aquatic food supply was 2.73 million metric tonnes (MMT) per year during the study period. We estimated that 0.62 MMT yr^−1^ or 22.7% of that total supply was physically lost or wasted (Fig. 1b), which is 43–55% less than older estimates for aquatic FLW in North America (50% FLW) or the United States (40–47% FLW)^2,30^. Older estimates were based on an incomplete model of the supply chain and sparse data with many simplifying assumptions (Supplementary Table 1). Similarly, recent FLW estimates for aquatic food in China (20%)^31^ are 43% lower than earlier FAO estimates^2^, which suggests that more detailed approaches tend to shrink loss estimates.

### FLW by stage of the supply chain

At all supply chain stages we estimated physical loss, which is products physically removed from the human food supply. In production, processing and distribution stages we also assessed quality loss, which corresponds to products sold at an economic loss that may remain in the human food supply or have other uses (for example, animal feed). Quality loss can occur when products are damaged during harvest or transport, not properly processed or packaged, contaminated by insects, or have time and temperature abuse^22^. Byproducts and inedible waste (that is, heads, frames, tails) that were not sold as human food were excluded from this analysis.

#### Overview

**Fig. 2:**
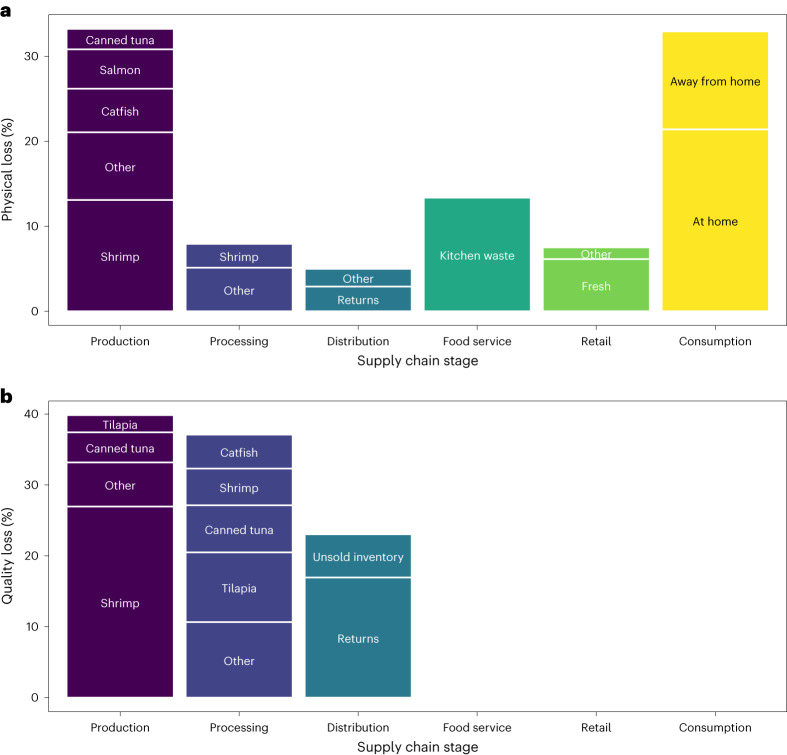
2014–2018 average share of FLW by stage of the US aquatic food supply chain. **a**,**b**, Reported for physical (**a**) and quality loss (**b**), and by source of loss. Columns represent the contribution of each supply chain stage to overall FLW (for example, all columns sum to 100%). Losses <2% of the total are combined in the ‘other’ categories. Catfish and *Pangasius* are combined into the catfish species group. Quality loss was not available for the food service, retail and consumer stages. Values are reported in Supplementary Data 2.

For physical loss, the production and consumption stages of the supply chain had the highest amounts of physical loss, each contributing one-third of total loss (Fig. 2a). Processors and distributors contributed 7.9% and 5.0% of total physical loss, respectively (Fig. 2a). Consumer-facing businesses contributed over one-fifth (20.9%) of overall food loss (7.5% retail + 13.4% food service; Fig. 2a). For quality loss, the production, processing and distribution stages were 41%, 37% and 23% of overall quality loss measured in the system, respectively (Fig. 2b); however, quality loss data were not available for the retail, food service or consumer stages.

#### Production

At the capture fishery and aquaculture production stage, we collected primary and secondary data on the top ten species groups consumed in the United States and extrapolated these findings to the total aquatic food supply (Fig. 1a). The total physical loss of edible aquatic food was 0.21 MMT yr^−1^, based on a physical loss rate of 7.5% (Fig. 1b). Quality loss was 0.09 MMT yr^−1^ based on a quality loss rate of 3.4% (Fig. 1c). The physical and quality loss rates suggest that while producers can find markets for some of their lower-quality products, by discounting instead of discarding, a notably large share of products is still removed from the human food supply, meriting further exploration. The species groups with the greatest contribution to production losses were shrimp, catfish, salmon, canned tuna and tilapia (Fig. 2a,b), which are the top-five most consumed species in the United States^32^.

Capture fisheries and aquaculture had physical loss rates of 5.9% and 8.2%, respectively, and their respective quality loss rates were 2.6% and 3.8% (Supplementary Table 5). Differences between loss rates of fisheries and aquaculture can be attributed to different assumptions we used for calculating harvest-stage mortalities. As aquaculture has more control over the production process^33^ and ideally all harvestable-sized individuals would go to market, we counted all mortalities of harvestable-sized animals as food loss. For capture fisheries, we did not assume that mortalities of theoretically harvestable individuals represent food loss because these mortalities occur in nature and are not easily tracked or managed. Therefore, in the capture fishery production stage we only include animals harvested and discarded as food loss. Given this definition difference, one cannot conclude that aquaculture is more wasteful, but rather that there are different levers for how loss can be better controlled.

#### Processing

Processing losses were calculated in a similar manner as production losses for the top ten species consumed in the United States and extrapolated to the US supply (Fig. 1a). Losses include mishandling, damage, disease, floor drops, quality loss and import refusals. Total processing losses were 0.05 MMT yr^−1^ for physical loss based on a physical loss rate of 1.8% (Fig. 1b). Quality loss was 0.09 MMT yr^−1^ based on a quality loss rate of 3.1% (Fig. 1c). Processors’ quality loss rate was much higher than physical loss rate, which suggests that processors have been effective at minimizing loss of human-edible food and are able to find markets for lower-quality products instead of discarding them. The species groups with the largest share of loss in processing were shrimp, tilapia, canned tuna and catfish (Fig. 2a,b).

#### Distribution

Distributors wasted the least amount of aquatic foods, a finding that agrees with previous work^34^. Distributors move large volumes of product on a daily basis, and fresh/live products spoil quickly; they have a strong incentive to minimize waste because aquatic foods are relatively expensive. Physical loss was 0.03 MMT yr^−1^ based on a physical loss rate of 1.2% (Fig. 1b). Physical losses were split among returns (60%), unsold inventory (35%) and food safety recalls (5%). Food safety recalls are important to control foodborne disease and allergen exposures but play a small role in food loss. Quality loss was 0.05 MMT yr^−1^ based on a quality loss rate of 2.0% (Fig. 1c). Quality losses came from either product returns (69%) or unsold inventory (31%). Communicating roles, preferences and expectations between supply chain members is critical for food safety^35^ and could also reduce product returns.

#### Retail and food service

Aquatic FLW in the US retail sector (that is, grocery stores) is well studied in the industry, while the food service sector (that is, restaurants and other commercial kitchens) has significant knowledge gaps that we filled using values from the literature (*n* = 13 studies). To estimate aquatic food losses across the US retail sector, we combined existing national retail surveys^36–38^ and a national retail sales database^7^. We estimated total physical losses of 0.05 MMT yr^−1^ based on a physical loss rate of 2.9% (Fig. 1b). The largest share of loss at retail was from discards of fresh products (81%) followed by frozen products (11%) and shelf-stable products (8%). Physical losses in food service kitchens were 0.08 MMT yr^−1^ based on a physical loss rate of 9.9% (Fig. 1b). FLW at the retail and food services stages is probably influenced by what species are offered, which depends on the type of outlet^39^ and consumer demographics^40^.

#### Consumption

Consumers contribute one of the largest shares of overall FLW, second only to production (Fig. 2a). This finding agrees with other studies suggesting consumers in high-income countries have high waste rates^31^. Nearly two-thirds (65%, 0.13 MMT yr^−1^) of consumer-level waste came from at home meals, with the remaining waste (35%, 0.07 MMT yr^−1^) attributed to away-from-home meals (Figs. 1b and 2a).

### FLW by species group

**Fig. 3:**
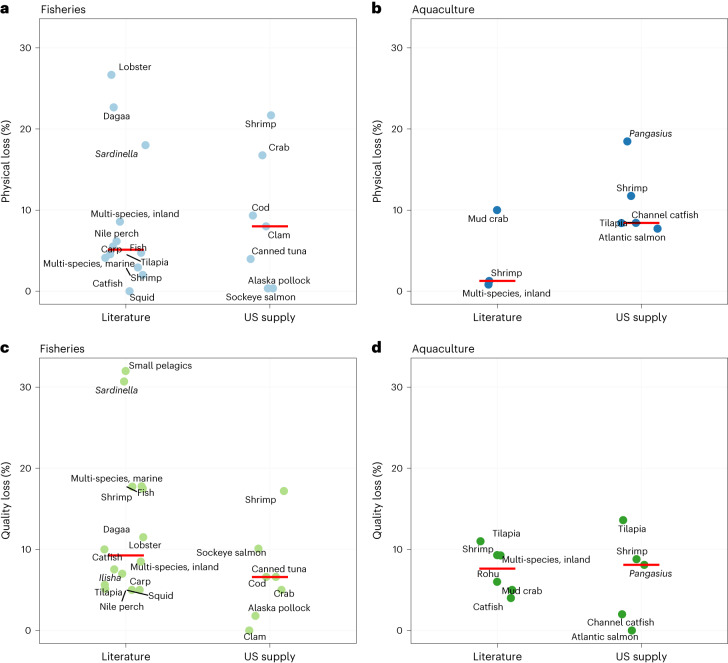
Physical and quality loss rates in capture fisheries and aquaculture for the combined production and processing stages. **a**–**d**, Rate of physical (**a**,**b**) and quality loss (**c**,**d**) in capture fisheries (**a**,**c**) and aquaculture (**b**,**d**) for the combined production and processing stages. Red bar is the median. Species <1% of US supply were removed. No supply cutoff was applied to values from the literature, which include species caught and consumed outside the United States. Values are reported in Supplementary Data 3.

Losses at the production and processing stages were analysed by species group and compared to literature values (Fig. 3). Capture fisheries have a wide range of losses (Fig. 3a,c) due to the diversity in species and gear types, as well as different levels of infrastructure, technology, capacity and governance in fishing regions^22,41^. The highest rates of physical loss were spiny lobster in Indonesia (27% loss); dagaa in Kenya, Tanzania and Uganda (23%); global shrimp (22%); and *Sardinella* spp. in Ghana (18%; Fig. 3a and Supplementary Fig. 2). The highest rates of quality loss in the literature were for small pelagics in Indonesia (32%); *Sardinella* spp. in Ghana (31%); ‘fish’ in Burkina Faso, Ghana and Togo (18%); and marine fish (hilsa, pomfret, lakkah and so on) in Bangladesh (18%; Fig. 3c and Supplementary Fig. 1). The Alaska pollock fishery in the United States, a purse seine fishery with industrial processing methods and low rates of discards, had among the lowest rates of loss (0.35% physical loss, 1.8% quality loss).

In aquaculture, the highest rates of physical loss were found in *Pangasius* in Vietnam (18%), shrimp in Vietnam (12%) and tilapia in China (11%; Fig. 3b and Supplementary Fig. 1). The highest rates of quality loss came from the literature: tilapia in China and Bangladesh (14% and 11% loss, respectively), shrimp in Bangladesh (9%), and freshwater species (carps, rui, catla) in Bangladesh (9%; Fig. 3d). Median quality loss rates were fairly similar between fisheries and aquaculture (Fig. 3c,d). This makes sense, given that processing methods are relatively similar across fisheries and aquaculture sectors.

More work is needed to characterize losses in aquaculture species that contribute half of the aquatic food supply^5^. Large knowledge gaps remain for losses in many regions of the world outside Africa and Asia (Supplementary Fig. 2). Additionally, more work is needed on gender and food loss. Women account for approximately half of the workforce in aquatic food value chains^5^, and it has been recognized that gender and gender equity affect loss^42–44^.

### Reasons for FLW and interventions

#### Capture fisheries

Losses in capture fisheries were mainly fish caught and discarded at sea rather than being landed. Discards are typically driven by market pressures or regulatory constraints. Catch may be discarded because it is inedible, has low or no economic value due to species, size or damage, or is prohibited by law due to catch quota or restrictions^45^. While discards have declined dramatically over the past four decades, certain gear types such as bottom trawl still have large impacts on natural resources and ecosystems^46^, and also on food loss. Fisheries managers should encourage selective fishing gear and the use of low-waste gears^47,48^, and can use food loss as an additional reason for their use (Table 1).

**Table 1:** Causes of aquatic FLW and key interventions

Supply chain stage	Causes of FLW	Relative contribution	Key interventions
Production	Fisheries: discards and bycatch Aquaculture: mortality of harvestable-sized animals Both: quality loss associated with damage/injury during harvest and post-harvest handling	High	Fisheries: adoption of selective fishing gears, improved cold chain and handling Aquaculture: improvements in aquatic animal health, farm management and hatchery genetics
Processing	Mishandling, damage, disease, floor drops, import refusals, quality loss, quality standards	Medium	Increased utilization of byproducts and sourcing of markets for lower-quality and niche products, utilization of frozen aquatic foods
Distribution	Returns (self and customer returns), unsold inventory, food safety recalls, quality loss	Low	Improved logistics and communication between supply chain actors, discounting strategies, improved cold chain
Retail	Unsold inventory, quality loss	Medium	Inventory management and discounting strategies, improved staff training and proper storage
Food service	Kitchen waste, unsold inventory	Medium	Inventory management, proper storage, promotional strategies, pre-portioned food, shelf-stable or frozen food
Consumption	At home: plate and household waste, aspirational shopping, discards due to spoilage or odour Away from home: plate waste, over-ordering, food sent back to the kitchen	High	Industry: innovations in processing and packaging that extend shelf life, shorter supply chains, shift to shelf-stable or frozen aquatic food, smaller portions in restaurants Consumers: educate consumers on improved purchase, handling, storage and preparation of aquatic food; promote purchasing of shelf-stable or frozen aquatic food

Identified through quantitative and qualitative data collection methods and literature.

#### Aquaculture

Losses in aquaculture were primarily from mortality of harvestable-sized animals, which was modelled for farmed channel catfish, *Pangasius*, tilapia, Atlantic salmon and shrimp (Supplementary Fig. 3), and extrapolated to the remainder of the US aquaculture supply. Mortalities are not typically considered a source of food loss in aquatic food production; however, they should be seen as such, particularly within aquaculture, because significant resources, including feed, are expended on the fish before it dies. Disease is a major cause of mortality on fish farms and a constraint for growth in the aquaculture sector, and the ongoing efforts to improve aquatic animal health could reduce these losses^49–51^ (Table 1).

Aquaculture producers we interviewed, including catfish farmers in the United States, *Pangasius* and shrimp farmers in Vietnam, and Atlantic salmon farmers in Norway, described concerns about diseases and water quality, and many explained that a disease outbreak can result in losing an entire pond, tank or pen of animals. A catfish farmer stated that monitoring and controlling aeration and water quality is critical for catfish producers. *Pangasius* farmers reported lower survival compared with a decade earlier and the reasons they identified were poor water quality, disease outbreaks and low quality of fingerlings. *Pangasius* farmers use Mekong River water in their ponds, and many expressed concern about pesticide contamination from nearby rice farms; they see improved regional planning and cooperation, led by government officials, as an important strategy to improve water quality. Some Atlantic salmon farmers in Norway explained that nearby salmon farms can negatively impact water quality and fish health, and they would also like to see improved coordination in the region to improve water quality and prevent disease outbreaks.

Origin and trade are additional factors to consider regarding FLW and are of particular importance for aquatic food. Aquatic foods as a category have the highest share of production traded^17^, and the majority of aquatic food in the US supply is imported from many countries^29^. The high share of imports differentiates it from most foods in the US supply (Supplementary Fig. 4). We disaggregated the US supply by production methods (capture fisheries, aquaculture) and origin (imported, domestic) and generated loss estimates for each group. At the production stage, imports made up 78–81% of production losses (Supplementary Fig. 5a), and more than a quarter (26%) of all FLW for the entire supply chain, which indicates that multinational efforts are needed to address FLW in the US food system. The share of losses from imports was higher than the share of imports in the US food supply (72% imported; Supplementary Fig. 5a), in part because imports are dominated by aquaculture, which has a higher rate of loss than capture fisheries (Supplementary Fig. 5b,c). LMICs also have higher rates of FLW compared with high-income countries in upstream stages of supply chains, which we also observed, which can be addressed with improvements in technology, infrastructure, and capacity building^22,31,41^ and trade incentives such as the US Seafood Import Monitoring Program, by targeting unsustainable fishing practices^52^.

#### Processing

Processors work closely with producers and other supply chain partners to increase the quality of their product. For example, in the Alaska sockeye salmon fishery, processors have incentivized fishers to deliver higher-quality fish by paying extra for chilled and bled fish and for using methods that reduce bruising of fish tissue. These practices result in higher-quality fish, which has allowed processors to shift away from selling canned salmon and instead sell fresh or frozen fillets at a higher price point. These shifts also have an unintended consequence that FLW is shifted towards the consumer, because fresh products have higher rates of FLW than canned products at the retail and consumer levels. One strategy to focus on quality while maintaining lower rates of FLW is to sell frozen fish (Table 1).

A major challenge and opportunity in the processing sectors is for businesses to create value from quality losses and byproducts (Table 1), which fits within the goal of a ‘circular economy’^53,54^. Processors’ own quality standards contribute to quality loss; but how those losses are handled differs widely across species, providing useful lessons on ways to reduce FLW. For example, farmed *Pangasius* processors in Vietnam and farmed salmon processors in Norway used trimmings for value-added products (that is, salmon burgers, human-grade fish oil). Processors in Vietnam had lower labour costs and turnover, which allowed for specialized processing (that is, removing fish swim bladders by hand), so that less-valuable co-products were kept in the human food supply. Interviewees in the sockeye salmon supply chain in Bristol Bay, Alaska, noted that utilization of byproducts was a challenge, mostly due to the short, intense fishing season and isolated geographic location. Sockeye salmon are caught during a few weeks in July, and processors explained that all of their resources, including labour and cold storage, are dedicated to the most valuable parts of the fish. Some sockeye salmon processors convert byproducts into fishmeal and oil, but many others grind and dump byproducts into the sea. Interviewees stated that if the fishery operated more months of the year, plants that would use all of the byproducts would have been built in the area; however, high shipping costs for remote regions such as Alaska remain a challenge. Farmed Atlantic salmon and other aquacultured species can be harvested and processed throughout the year.

US catfish processors shared that they have invested in automation, in part due to difficulties hiring and retaining staff. Cutting fish properly for optimal yield is a top priority for processors and can be a challenge when labour is in short supply and there is high turnover. Harvested catfish vary in size, though, and this results in off-sized fish being diverted to rendering plants to be turned into animal feed because automated processing equipment cannot accommodate them. Importantly, interviews with catfish processors took place before the COVID-19 pandemic, which demonstrates existing labour challenges in some aquatic food supply chains even before the disruptions resulting from the pandemic. In Vietnam, processing is done by hand and workers can process fish of various sizes. We also observed differences in quality standards that affected food loss. Low-quality and undersized farmed shrimp and *Pangasius* in Vietnam were sold at a discount to local markets, which kept these products in the human food supply, while in the United States, high quality standards for live products for certain species meant these products were discarded at a higher rate.

#### Distribution

Strategies to prevent FLW that interviewees described included constant communication between different teams in the company (for example, buyers, logistics, sales), discounting products that are not selling in a timely manner (including potentially taking a loss to avoid no sale) and requiring pre-orders for highly perishable and/or valuable products so they have a customer before they order the product.

#### Retail

A variety of strategies are used to reduce losses in retail settings as described by representatives of retail chains. Grocery chain interviewees described the importance of staff training, proper storage and ordering protocols to minimize losses in the seafood department (Table 1). Their focus was on reducing losses of fresh aquatic food, which is more valuable and lost at higher rates than frozen and canned aquatic food, which agrees with previous studies^7,55^. Some retail chains allow seafood managers to order products, others rely on historical sales data, but seafood department managers sometimes override the system because they do not want to run out of aquatic food products. ‘Blind ordering’ or ordering new products before previous orders have been delivered was detrimental to accurate ordering and waste prevention.

The two retail chains interviewed differ regarding discounting aquatic food. One chain uses markdowns to sell aquatic food that was not selling quickly. The other company does not use markdowns because they do not want to give customers the impression that they are selling low-quality aquatic food. Instead, they initially put the product in a larger package and if it does not sell they cut it and put it out the next day in smaller packages. The interviewee from this company also shared that having a well-stocked seafood department was a priority for the grocery store chain, even at the expense of FLW, because running out of a particular product would negatively impact the customer shopping experience.

#### Food service

Aquatic food products have higher price points in food service, so preventing FLW was seen as important by interviewees in the restaurant industry. They identified frequent ordering and deliveries, proper storage, inventory management, a waste log and cross utilization of aquatic food products as important for reducing FLW (Table 1). More specifically, staff are instructed to inspect aquatic food when it is delivered to make sure there are no quality issues, store some highly perishable items in ice, use a store inventory to encourage use of older foods first, record waste of high-value items (including aquatic foods) on a log with the reason so recurring issues can be addressed and plan to use trimmings in another dish on the menu (for example, seafood salad, stew). Interviewees explained that ensuring staff consistently maintain efforts in these areas was difficult in busy commercial kitchens and has become more difficult due to staffing shortages and high turnover resulting from the COVID-19 pandemic. Additional strategies mentioned less frequently included regular maintenance of walk-in refrigerators and freezers to avoid breakdowns, educating servers so they can market aquatic food to customers, and creating a ‘chef’s special’ to sell certain products more quickly. Some food service companies create value with larger portion sizes^56^; however, this can lead to overeating or plate waste (for example, uneaten food) by consumers^57^.

Retailers and food service businesses described the tension between optimizing freshness and labour efficiency. The timing and extent of fish processing is critical for fresh aquatic food. Cutting fish into fillets and other products at processing plants before it arrives at stores/restaurants is more efficient from a labour standpoint, because processing plants have specialized machinery and workers to process a large quantity of fish. Staff at stores and restaurants have many other duties, and training is needed to build cutting skills, especially due to the variety of fresh aquatic food at some stores and restaurants. However, shipping minimally processed fish as far along in the supply chain as possible is an important strategy for maintaining freshness and extending shelf life for downstream consumers. Shipping whole fish also has the unintended consequence of decentralizing the waste stream, which makes rendering byproducts less feasible from a logistics standpoint.

#### Consumption

The majority of aquatic foods in the United States are purchased at retail and consumed at home^18^, which explains the larger amount of FLW attributed to food at home. We used household food diaries to estimate a physical loss rate of 8.5% at home. Others have used bin digs or scales in high-income country households to identify physical loss rates of 7% for fish, meat and eggs; 9.6% for fish; and 13.2% for fish and meat^58–60^. By contrast, indirect methods have estimated much higher physical loss rates of 17% for most types of canned fish and shellfish, and 40% for fresh and frozen fish and shellfish, which was calculated by comparing national estimates of retail sales to national estimates of dietary intake^19^. Household-level studies using scales to measure food waste are preferred for quantitative estimates; however, there is no definitive study of household-level FLW in the United States and accurately accounting for household waste remains challenging.

Our survey research found that the most common reported reasons for throwing out aquatic foods were ‘did not want as leftovers’ (18%) and ‘bought too much’ (16%). Slimy appearance and odours each accounted for over 6% of wasted aquatic food. The top motivation for reducing discards of aquatic food was saving money (64%). Prior research with aquatic food consumers suggested that many considered themselves less likely to waste aquatic food than other foods due to its relatively high price and their taste preferences for it^55^.

The large contribution of consumers to overall FLW indicates opportunity to reduce FLW through targeted interventions at the consumer level. The relatively low proficiency of consumers in handling, storing and preparing aquatic food may lead to consumers prematurely throwing aquatic food away due to concerns with safety or quality, and is an opportunity to educate them. In addition, shorter supply chains and innovations in processing, packaging and preservation techniques can extend the shelf life of aquatic food products and reduce at-home aquatic food waste (Table 1).

## Discussion

As food security, economic and sustainability challenges grow^3,29,61,62^, strategies to reduce losses and waste of economically valuable^61,62^ and healthy aquatic food products^3^ become ever-more essential. This mixed methods analysis provides a comprehensive assessment of US aquatic FLW, estimating that 22.7% of the national aquatic food supply is lost or wasted. The highest loss and waste occurred at production and consumption stages, with considerable variation by species, geography and production method. The findings align with prior estimates of FLW more broadly^31,63^; by contrast, prior modelling of FLW in aquatic food had estimated far higher losses based on limited data and flawed assumptions regarding the importance of aquaculture, trade, food service and consumer patterns^2,30^. While the findings reflect the US supply, the sourcing is largely from imports and thus has global implications. These estimates take a US supply chain perspective and what is considered ‘edible’ by US consumers. We did not consider losses from parts of the fish that US consumers consider ‘inedible’, or fish that could be consumed by humans but are used as animal feed^64,65^.

Estimates of aquatic FLW serve multiple functions. Businesses throughout the supply chain may draw insights relevant to improving their own operations and tracking their discards and quality losses more thoroughly. Third-party auditors could add FLW targets to certification schemes. The data can be shared to improve aquatic food estimates within existing databases such as through ReFED (https://refed.org/food-waste/the-challenge/), the US Department of Agriculture’s Loss-Adjusted Food Availability data series and the FAO. The findings also provide a benchmark to track progress within the fisheries and aquaculture sectors, and the methodology can be applied to other regions or food sectors.

The findings highlight several important dilemmas and tradeoffs in addressing aquatic food FLW. For example, there are different views on when in the value chain is best for secondary processing (that is, processing a head-on/off gutted fish into a fillet) and how that affects quality, shelf life, waste and labour efficiency. We highlight the reality of conflicting objectives whereby preventing quality loss early in the supply chain (that is, producing higher-quality fish) led some companies to shift from selling lower-quality canned fish to higher-quality fresh or frozen forms, which shifts losses to later in the supply chain after additional resources are invested in distribution and storage. The research also distinguishes physical and quality losses; however, there is fluidity between these categories depending on local markets and preferences. For example, in Vietnam, catfish processing losses are discounted and sold as human food (that is, quality loss), but in the US, catfish processing losses are rendered into animal feed (that is, physical loss). Last, we note a methodological dilemma in comparing losses from aquaculture and capture fishery production. The higher estimate for aquaculture results from inclusion of mortalities; however, we did not treat mortalities of unharvested wild fish as loss, being both uncounted and outside the control of producers.

Finally, the findings and qualitative insights inform prioritization of solutions, including identifying areas of need for innovation, government incentives, policy support, infrastructure and equity. Based on this analysis, we highlight the following opportunities and needs. First, aquatic food production, particularly aquaculture, and home consumption have the largest waste footprint and should be prioritized for solutions. Water quality, disease prevention strategies, improved hatchery genetics and governance can reduce mortality in aquaculture. For capture fisheries, priorities include harvesting methods to reduce unwanted catch and improved cold chain and handling of fish. We previously described solutions relevant to consumer discards, which include proficiency with preparing fish, perceptions and knowledge about aquatic food, perishability, and planning^55^. The food processing sector performs well in waste reduction and in upcycling trimmings; methods should be further disseminated and analogous practices explored in other sectors. LMICs need further investment in capacity, infrastructure and technology to enable improved waste reduction. Gender-sensitive and -transformative approaches may be necessary in some contexts to reduce FLW. In addition, improved and ongoing data collection regarding aquatic food waste within and across sectors and supply chains will improve action efforts. FLW research and surveillance should segment aquatic food from meats when feasible. Broader incentives to reduce aquatic FLW may be derived both from the lost value and potentially, incorporation of waste metrics into sustainability monitoring and consumer labelling. Multiple other approaches to reducing aquatic FLW are being applied in real-world settings globally^21^ and further study is needed to assess impacts.

## Methods

### Scope, boundary conditions and terms

This study estimated loss and waste in the US aquatic food supply chain from 2014 to 2018, beginning at the production stage and ending when aquatic foods were consumed in the United States or removed from the supply chain. The boundary conditions follow recommendations made by the FAO framework^13^. At the production and processing stages, we selected the top ten species groups in the US supply for analysis (Fig. 1a), which represents 89% of US aquatic food supply^32^, including both capture fisheries and aquaculture production methods. In subsequent stages (distribution, retail, food service and consumption stages), we collapsed all aquatic foods together into a single category for ease of tracking product flows (Fig. 1a). After the distribution stage we split the aquatic food supply into (1) products sold at retail (that is, supermarkets) and consumed at home, and (2) products sold at food service (that is, restaurants and institutions) and consumed away from home (Fig. 1a).

Data on physical losses, which are food products physically removed from the human food supply, were collected from all stages of the supply chain. Quality losses were also collected in the production, processing and distribution stages. Quality losses are edible products sold at a discount or donated (for example, imperfections, not meeting quality standards and so on), but not necessarily removed from the human food supply. We excluded byproducts and inedible waste (that is, heads, frames, tails) that are sold for animal feed or pet food.

### FLW quantitative data collection

#### Production and processing

Production and processing loss data were collected using surveys, semi-structured qualitative interviews and literature for the top ten species groups consumed in the United States (shrimp, canned tuna, salmon, Alaska pollock, tilapia, catfish, *Pangasius*, crab, cod, clams) and all other species combined in an ‘other’ category (Supplementary Table 1).

To fill key data gaps we collected primary data in seven sectors that are important for the US aquatic food supply, but currently lack FLW estimates. These sectors were Vietnam farmed shrimp (*Penaeus monodon*, *Litopenaeus vannamei*); Vietnam farmed *Pangasius* (*Pangasius hypophthalmus*); southern US farmed channel catfish (*Ictalurus punctatus*) and hybrid catfish (*I. punctatus* × *I. furcatus*); Norway farmed Atlantic salmon (*Salmo salar*); US Alaska wild capture sockeye salmon (*Oncorhynchus nerka*); US wild capture Alaska pollock (*Gadus chalcogrammus*); and canned tuna from the Pacific tuna fisheries (*Thunnus* spp.). Businesses within these seven sectors were recruited through trusted intermediaries and industry contacts. Overall, *n* = 24 producers and *n* = 20 processors completed surveys on rates of physical and quality loss, such as discards, mortalities, oversized or undersized harvests, temperature abuse, damaged or decomposing products and other forms of loss (Supplementary Tables 5 and 6). Most of the primary quantitative data at these stages were collected in person in 2019.

To supplement the primary data collection and fill data gaps we conducted a non-systematic literature review. The literature search was performed in Google Scholar using a list of keywords. Relevant records were compiled, along with their reference lists, until an exhaustive list was collected. Records were screened and rates of physical and quality FLW were extracted from *n* = 33 studies, with *n* = 19 studies having usable data (Supplementary Data 2).

For aquaculture species, we estimated the biomass lost when harvestable-size animals died before harvest, and then calculated the edible fraction remaining as producer-level food loss (Supplementary Information). For imported species, we used import inspection data to calculate an import refusal rate as previously described^66^, which was added to processor losses.

After collecting loss data for each species group and production method in the study, we then applied two types of weighting factor to generate (1) species group loss estimates and (2) national loss estimates. First, to create species group loss estimates (that is, overall shrimp loss), we had to account for losses coming from multiple production methods (that is, aquaculture shrimp loss + wild-caught shrimp loss) and weight these losses by the share of supply coming from each production method. Developing weighting factors for production method (aquaculture versus wild capture) and origin (domestic versus imports) was performed using previously described methods^40^ (Supplementary Table 1 and Supplementary Information). For example, shrimp in the US supply comes 15% from capture fisheries and 85% from aquaculture, therefore aquaculture losses will have a greater contribution to overall shrimp loss. Second, to create a weighted national average we weighted the overall species group loss rates by the share each species group contributes to the US aquatic food supply. For example, shrimp makes up 26% of the US supply and was given that corresponding weight in the overall model. The species group and national weighted averages were performed for both the production and processing stages.

#### Distribution

We calculated a loss rate for all aquatic food distributed in the United States, which included losses at wholesale and transportation based on our survey and secondary data on national food safety recalls (Supplementary Table 7). The survey asked about wholesale and customer returns, and any unsold inventory that was removed from the human food supply (that is, sent to landfill or rendered), and foods that were donated or discounted for resale to humans. We assumed that distributors sold processed forms of aquatic food and did not adjust losses for edible yield. Four US and one Canadian business responded to the quantitative survey. These groups had total aquatic food sales of 33,000 tonnes per year. Loss estimates were calculated from a national food recall database provided by the US Food and Drug Administration, as previously described^66^. We summed all reported aquatic food recalls in the United States (1,400 tonnes per year) during the study period and divided them by the aquatic food supply to develop a rate of recalls. We assumed that food recalls were removed from the human food supply.

#### Retail

A national retail loss rate was developed using survey data about losses of fresh, frozen and canned aquatic food (Supplementary Table 7), and weighted by share of aquatic food sold as fresh, frozen or shelf-stable using nationally representative retail sales data^7^ (Supplementary Table 9). The loss estimates came from a survey of US grocery store chains conducted by the Food Marketing Institute, a trade association for the retail sector, and was conducted in 2014, 2016 and 2018, with a total of 90 responses. We assumed that retail businesses sold processed forms of aquatic food and did not adjust losses for edible yield. Loss rates for frozen and canned aquatic foods were not available, so all frozen and all shelf-stable foods were used as proxies, which we validated with individual chain retailers.

#### Food service

Estimates of food service losses for aquatic foods are limited to the literature as our recruitment of national seafood chain restaurants was unsuccessful. Following a non-systematic literature search, we extracted data from 13 peer-reviewed articles from eight countries (Canada, Finland, Malaysia, Portugal, Sweden, Switzerland, UK and United States). These studies were based on seven restaurants, five schools or universities, three food service businesses, one workplace, one daycare, one home food delivery service and one experimental feeding trial (some studies had multiple sites; Supplementary Tables 7 and 8). Of the 13 records, six had information on kitchen waste and 13 had information on consumer plate waste. We assumed that food service businesses used processed forms of aquatic food and did not adjust losses for edible yield. Few studies focused specifically on aquatic food, therefore entree, meat and all kitchen waste was used as a proxy. Quality loss was not available for food service or retail.

#### Consumer

At-home waste estimates were developed using our food diary survey of US aquatic food consumers (*n* = 70) conducted from 14 June to 15 July 2019 (Supplementary Table 7) and based on raw edible portions. Survey responses were weighted by income level to match those of aquatic food consumers, which skews higher than the national average, using nationally representative dietary intake data. Away-from-home waste was calculated using secondary data on consumer plate waste from food service meals as described above. To make the at-home and away-from-home waste rates nationally generalizable, we weighted these loss rates by the share of aquatic food consumed at home versus away from home using nationally representative dietary intake data^18^ (Supplementary Table 9).

#### Overall loss rate calculations

To estimate the quantity of food lost at each stage of the supply chain, we multiplied the rate of loss at that stage by the quantity of aquatic food available at that stage. Across the study, quantities at each stage were converted to raw edible weight. The total US supply was calculated by multiplying the average per capita aquatic food availability from 2014 to 2018, provided by the US National Marine Fisheries Service^67^, by the US population plus any pre-harvest losses. The overall loss rate for the US supply was calculated as the sum of all losses at each stage divided by the total US supply.

#### Sources of bias and error

There are several notable sources of bias and error to this modelling approach. First, bias was introduced in converting aquatic foods to different product forms. These conversions were needed to compare products within and across stages of the supply chain and required making assumptions about the types of product form at each stage. The approach also required assumptions about what is considered ‘edible’ in the US supply, which may not be true for all groups in the US or other countries. We assumed that products in the distribution, retail and food service, and consumer stages, were already processed into raw edible forms; however, some businesses and consumers purchase whole fish. To help counteract this potential source of bias, we asked respondents to report only losses of edible products and included a definition of edible and inedible products in the survey tool.

Second, error was introduced in estimating the share of products from aquaculture versus capture fisheries and imported versus domestic origin, because trade codes have broad product categories with a mixture of product forms. For example, the bivalve category includes a mixture of shell-on and shell-off products, which can affect the product weights dramatically and in ways that were not controlled.

Third, we assumed that the sectors in which we collected primary and secondary data were generalizable to all regions that produced aquatic foods for the US market. We attempted to control this source of bias by selecting sectors and regions for study that are large contributors to the US supply.

Fourth, we introduced bias in the distribution stage by oversampling specialty seafood wholesalers that sell live and fresh aquatic foods, and undersampled broadline distributors that sell canned and frozen aquatic foods. We were not able to weight the sample because there are no estimates for the share of US sales from broadline versus specialty seafood wholesale. This may skew distributor losses higher than normal because food loss is usually higher for fresh products; however, we do not anticipate this bias has a meaningful impact on the overall findings because the distributor stage had a small (5%) contribution to overall FLW.

Last, we were unable to collect primary data for the food service stage including consumers’ away-from-home waste, and instead relied on literature values. This introduced potential error and uncertainty because the literature was not specific to aquatic foods, and to increase our sample size we included estimates from outside the United States.

#### Application to other settings

The quantitative methods developed in this study can be applied to other settings with some modifications. The loss estimates are based on species groups commonly consumed in the United States. Weighting factors for production methods are also specific to the US supply, as are the weighting factors for the share of products distributed to retail versus food service and consumed at home versus away from home among US consumers. We also made assumptions about what is ‘edible’ by US consumers, which manifest in choices of edible yield values that may be different for other countries. Consumer waste is a large share of overall FLW in the model and we recommend using local consumer waste estimates where possible.

### FLW qualitative data collection

We conducted semi-structured qualitative interviews with business owners and operators in every stage of the supply chain to complement the quantitative data and provide a better understanding of perceptions, causes and trends in FLW, and current and potential strategies to reduce FLW. Interviews were conducted in person, over the phone, or via Zoom (Zoom Video Communications). A notetaker participated in each interview to accurately capture interviewee responses, and the data were analysed using MAXQDA (VERBI Software). Qualitative interviews for producers (*n* = 19) and processors (*n* = 14) were performed for wild-caught US sockeye salmon, farmed US catfish, farmed Norway Atlantic salmon, farmed Vietnam *Pangasius* and shrimp from 2019 to 2021. Additionally, we interviewed wholesale businesses (*n* = 4), retail chains (n = 2) and food service businesses (*n* = 5) in the United States in 2021.

### Ethics statement

The project was approved by the Institutional Review Boards at Johns Hopkins School of Public Health (IRB no. 8345) and University of Florida (IRB no. 201901559).

### Reporting summary

Further information on research design is available in the Nature Portfolio Reporting Summary linked to this article.

### Supplementary information

Supplementary Information Supplementary Figs. 1–5, Table 1–9 and Notes.

Reporting Summary

Supplementary Data 1 FLW rates by stage of the supply chain.

Supplementary Data 2 FLW rates by species and stage of the supply chain.

Supplementary Data 3 FLW rates at the production and processing stages combined, and comparison between this study and literature values.

## Data Availability

Data to produce Figs. 1–3 are available in the Supplementary Information.

## Code availability

Code to produce figures and tables is available upon request.

## References

[1] *Global Initiative on Food Loss and Waste Reduction* (FAO, 2015); https://www.fao.org/3/i4068e/i4068e.pdf

[2] Gustavsson, J., Cederberg, C., Sonesson, U., Van Otterdijk, R. & Meybeck, A. *Global Food Losses and Food Waste* (FAO, 2011).

[3] Golden CD (2021). Aquatic foods to nourish nations. Nature.

[4] Naylor RL (2021). Blue food demand across geographic and temporal scales. Nat. Commun..

[5] *The State of World Fisheries and Aquaculture 2022: Towards Blue Transformation* (FAO, 2022).

[6] Gram, L. in *Compendium of the Microbiological Spoilage of Foods and Beverages* (eds Sperber, W. H. & Doyle, M. P.) 87–119 (Springer, 2009).

[7] Love DC (2022). An overview of retail sales of seafood in the USA, 2017–2019. Rev. Fish. Sci. Aquac..

[8] Summers G, Wibisono RD, Hedderley DI, Fletcher GC (2017). Trimethylamine oxide content and spoilage potential of New Zealand commercial fish species. N. Z. J. Mar. Freshwater Res..

[9] IPCC *Climate Change 2022: Mitigation of Climate Change* (2022).

[10] Gu B, Zhang X, Bai X, Fu B, Chen D (2019). Four steps to food security for swelling cities. Nature.

[11] Springmann M (2018). Options for keeping the food system within environmental limits. Nature.

[12] West PC (2014). Leverage points for improving global food security and the environment. Science.

[13] *Definitional Framework of Food Loss* (FAO, 2014); https://www.fao.org/fileadmin/user_upload/save-food/PDF/FLW_Definition_and_Scope_2014.pdf

[14] Dou, Z. & Toth, J. D. Global primary data on consumer food waste: rate and characteristics – a review. *Resour. Conserv. Recycl*. **168**, 105332 (2021).

[15] Fanzo, J. et al. Rigorous monitoring is necessary to guide food system transformation in the countdown to the 2030 global goals. *Food Policy* **104**, 102163 (2021).

[16] Gustavsson, J., Cederberg, C., Sonesson, U. & Emanuelsson, A. *The Methodology of the FAO Study: “Global Food Losses and Food Waste - Extent, Causes and Prevention” - FAO, 2011* SIK Report No. 857 (Swedish Institute for Food and Biotechnology, 2013); https://www.diva-portal.org/smash/get/diva2:944159/FULLTEXT01.pdf

[17] Anderson JL, Asche F, Garlock T (2018). Globalization and commoditization: the transformation of the seafood market. J. Commod. Mark..

[18] Love DC (2020). Food sources and expenditures for seafood in the United States. Nutrients.

[19] Muth, M. K. *Consumer-Level Food Loss Estimates and Their Use in the Economic Research Service (ERS) Loss-Adjusted Food Availability Data (FAD)* (DIANE Publishing, 2011).

[20] *The State of Food and Agriculture 2019: Moving Forward on Food Loss and Waste Reduction* (FAO, 2019).

[21] *Food Loss and Waste Database* (FAO); https://www.fao.org/platform-food-loss-waste/flw-data/en/

[22] Kruijssen F (2020). Loss and waste in fish value chains: a review of the evidence from low and middle-income countries. Glob. Food Sec..

[23] Asche F (2022). China’s seafood imports - not for domestic consumption?. Science.

[24] Hoover, D. & Moreno, L. *Estimating Quantities and Types of Food Waste at the City Level* (Natural Resources Defense Council).

[25] Roe BE, Apolzan JW, Qi D, Allen HR, Martin CK (2018). Plate waste of adults in the United States measured in free-living conditions. PLoS ONE.

[26] Conrad Z (2018). Relationship between food waste, diet quality, and environmental sustainability. PLoS ONE.

[27] Johnson, A., McDermott, C., Elliott, D., Hunter, K. & De Venecia, C. *2017 Oregon Wasted Food Study: Residential Sector Waste Sort, Diary, and Survey Study: Summary of Findings* (Community Environmental Services, Portland State University, 2018).

[28] Buzby, J. C., Farah-Wells, H. & Hyman, J. *The Estimated Amount, Value, and Calories of Postharvest Food Losses at the Retail and Consumer Levels in the United States* USDA-ERS Economic Information Bulletin No. 121(USDA-ERS, 2014); 10.2139/ssrn.2501659

[29] Gephart JA (2021). Environmental performance of blue foods. Nature.

[30] Love DC, Fry JP, Milli MC, Neff RA (2015). Wasted seafood in the United States: quantifying loss from production to consumption and moving toward solutions. Glob. Environ. Change.

[31] Xue L (2021). China’s food loss and waste embodies increasing environmental impacts. Nat. Food.

[32] Top 10 list offers a look back in time. *NFI Media* (16 May 2022); https://aboutseafood.com/press_release/top-10-list-offers-a-look-back-in-time/

[33] Asche F, Smith MD (2018). Induced innovation in fisheries and aquaculture. Food Policy.

[34] Dong W (2022). A framework to quantify mass flow and assess food loss and waste in the US food supply chain. Commun. Earth Environ..

[35] Love DC (2020). Performance and conduct of supply chains for United States farmed oysters. Aquaculture.

[36] *The Food Retailing Industry Speaks 2015* (Food Marketing Institute, 2015).

[37] *The Food Retailing Industry Speaks 2017* (Food Marketing Institute, 2017).

[38] *The Food Retailing Industry Speaks 2019* (Food Marketing Institute, 2019).

[39] Love DC (2021). Nutrition and origin of US chain restaurant seafood. Am. J. Clin. Nutr..

[40] Love DC (2022). Affordability influences nutritional quality of seafood consumption among income and race/ethnicity groups in the United States. Am. J. Clin. Nutr..

[41] Affognon H, Mutungi C, Sanginga P, Borgemeister C (2015). Unpacking postharvest losses in sub-Saharan Africa: a meta-analysis. World Dev..

[42] Nordhagen, S. *Gender Equity and Reduction of Post-Harvest Losses in Agricultural Value Chains* (GAIN, 2021); https://www.gainhealth.org/sites/default/files/publications/documents/gain-working-paper-series-20-gender-equity-and-reduction-of-post-harvest-losses-in-agricultural-value-chains.pdf

[43] Kaminski AM (2020). Fish losses for whom? A gendered assessment of post-harvest losses in the Barotse Floodplain Fishery, Zambia. Sustain. Sci. Pract. Policy.

[44] Cole SM (2020). Gender accommodative versus transformative approaches: a comparative assessment within a post-harvest fish loss reduction intervention. Gend. Technol. Dev..

[45] Zeller D, Cashion T, Palomares M, Pauly D (2018). Global marine fisheries discards: a synthesis of reconstructed data. Fish Fish..

[46] Gilman E (2020). Benchmarking global fisheries discards. Sci. Rep..

[47] Enever R, Revill AS, Grant A (2009). Discarding in the North Sea and on the historical efficacy of gear-based technical measures in reducing discards. Fish. Res..

[48] Jenkins LD, Garrison K (2013). Fishing gear substitution to reduce bycatch and habitat impacts: an example of social–ecological research to inform policy. Mar. Policy.

[49] Patil PK (2021). Economic loss due to diseases in Indian shrimp farming with special reference to *Enterocytozoon hepatopenaei* (EHP) and white spot syndrome virus (WSSV). Aquaculture.

[50] Stentiford GD (2017). New paradigms to help solve the global aquaculture disease crisis. PLoS Pathog..

[51] Bondad-Reantaso MG (2005). Disease and health management in Asian aquaculture. Vet. Parasitol..

[52] Fang Y, Asche F (2021). Can U.S. import regulations reduce IUU fishing and improve production practices in aquaculture?. Ecol. Econ..

[53] Donner M, Verniquet A, Broeze J, Kayser K, De Vries H (2021). Critical success and risk factors for circular business models valorising agricultural waste and by-products. Resour. Conserv. Recycl..

[54] Teigiserova DA, Hamelin L, Thomsen M (2020). Towards transparent valorization of food surplus, waste and loss: clarifying definitions, food waste hierarchy, and role in the circular economy. Sci. Total Environ..

[55] Neff, R. A. et al. Consumer seafood waste and the potential of a ‘direct-from-frozen’ approach to prevention. *Foods* **10**, 2524 (2021). 10.3390/foods10112524 PMC8618751 34828809

[56] McAdams B, von Massow M, Gallant M, Hayhoe M-A (2019). A cross industry evaluation of food waste in restaurants. J. Foodserv. Bus. Res..

[57] Vizzoto F, Testa F, Iraldo F (2021). Strategies to reduce food waste in the foodservices sector: a systematic review. Int. J. Hosp. Manag..

[58] Silvennoinen K, Katajajuuri J-M, Hartikainen H, Heikkilä L, Reinikainen A (2014). Food waste volume and composition in Finnish households. Br. Food J..

[59] Quested, T. & Murphy, L. *Household Food and Drink Waste: A Product Focus* (Wastes & Resources Action Programme, 2014).

[60] Ventour, L. *The Food We Waste* (Wastes & Resources Action Programme, 2008).

[61] Conrad Z (2020). Daily cost of consumer food wasted, inedible, and consumed in the United States, 2001–2016. Nutr. J..

[62] de Visser-Amundson, A. & Kleijnen, M. in *Food Waste Management: Solving the Wicked Problem* (eds Närvänen, E. et al.) 57–87 (Springer, 2020).

[63] *Food Waste Challenge* (ReFed); https://refed.org/food-waste/the-challenge/

[64] Vianna GMS, Zeller D, Pauly D (2020). Fisheries and policy implications for human nutrition. Curr. Environ. Health Rep..

[65] Cashion T, Le Manach F, Zeller D, Pauly D (2017). Most fish destined for fishmeal production are food-grade fish. Fish Fish..

[66] Love DC (2021). Risks shift along seafood supply chains. Glob. Food Sec..

[67] *Fisheries of the United States, 2019* (National Marine Fisheries Service, 2021); https://media.fisheries.noaa.gov/2021-05/FUS2019-FINAL-webready-2.3.pdf?null=

