# Supplementary material for: Aquatic food loss and waste rate in the United States is half of earlier estimates
Source: Nat Food. 2023 Dec 13;4(12):1058–69. doi: 10.1038/s43016-023-00881-z (PMC10727981; doi:10.1038/s43016-023-00881-z)
Supplement: Supplementary file 1 — Supplementary Figs. 1–5, Table 1–9 and Notes. [file 43016_2023_881_MOESM1_ESM.pdf]

nature food

Article https://doi.org/10.1038/s43016-023-00881-z

Aquatic food loss and waste rate in the United States is half of earlier estimates

In the format provided by the authors and unedited

SUPPLEMENTARY INFORMATION

Comparison to the literature 2

Supplementary Table 1. Physical loss rates for aquatic foods in this study compared to similar estimates in the literature. 2

Supplementary Fig. 1. Loss of aquatic foods by species for the production and processing stages combined. Values reported in Supplementary Data 3. 3

Supplementary Fig. 2. Loss of aquatic foods by region for the production and processing stages combined. Red bar at median. Values reported in Supplementary Data 3. 4

Estimating production method and origin of aquatic food groups 5

Supplementary note 5

Supplementary Table 2. Weighting factors for the US aquatic food supply. 6

Modeling growth and biomass loss in aquaculture 7

Supplementary note 7

Supplementary Table 3. Parameters used to estimate growth and biomass loss at the grow-out stage. 8

Supplementary Table 4. Biomass loss ratios for five species under three scenarios. 8

Supplementary Fig. 3. Growth, survival, biomass, and biomass loss modeled for five aquaculture species under three scenarios. 9

Origin of fisheries and aquaculture products and food loss 10

Supplementary Fig. 4 Import share of U.S. food consumption, 2011-2013. [63] 10

Supplementary Fig. 5. The United States aquatic food supply, physical loss, and quality loss by production method and origin. Losses < 2% of the total combined in "other" categories. (a) Origin: domestic vs imported. (b) Production method: fisheries vs aquaculture. (c) Origin and production methods combined. Within parts (a), (b), and (c); all columns of the same color sum to 100%. 11

Estimates of loss and waste in United States aquatic food supply chains 12

Supplementary Table 5. Production loss in the United States aquatic food supply. 12

Supplementary Table 6. Processing loss in the United States aquatic food supply. 14

Supplementary Table 7. Distribution, retail, food service and consumer loss in the United States aquatic food supply. 16

Supplementary Table 8. Literature values for food service kitchen waste and plate waste. ... 17

Supplementary Table 9. Weighting factors for aquatic food supply chains. 18

References 19

Comparison to the literature

Supplementary Table 1. Physical loss rates for aquatic foods in this study compared to similar estimates in the literature.

Stage	United States (this study, 2014-2018)	North America [4] (2007)	United States [5] (2009-2013)
Production	7.5%	-	-
Aquaculture	8.2%	-	-
Fisheries	5.9%	12%	17% (US); 8% (imports)
Processing	1.8%	-	-
Aquaculture	1.7%	-	-
Fisheries	1.7%	6%	6%
Distribution	1.2%	-	-
Retail	2.9%	-	-
Fresh	6.7%	9%	9%
Frozen	0.7%	-	-
Canned	1.0%	5% (processed)	5% (processed)
Food Service	9.9%	-	-
Consumption	8.8%	-	-
At home	8.5%	33% (fresh); 10% (processed)	40% (fresh); 17% (processed)
Away from home	9.4%	-	-
Overall	22.7%	50%	40-47%

(-) not available

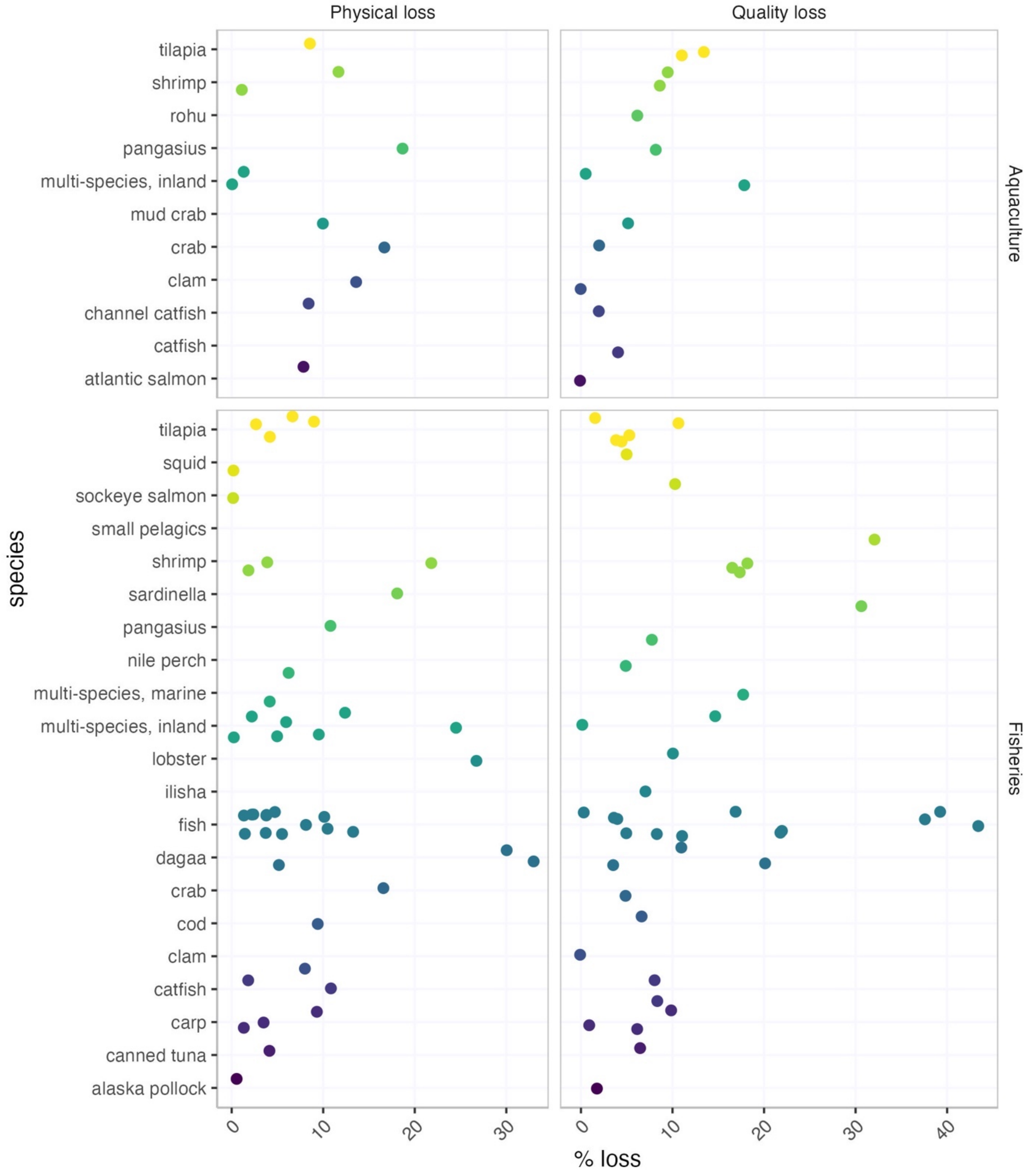

Supplementary Fig. 1. Loss of aquatic foods by species for the production and processing stages combined. Values reported in Supplementary Data 3.

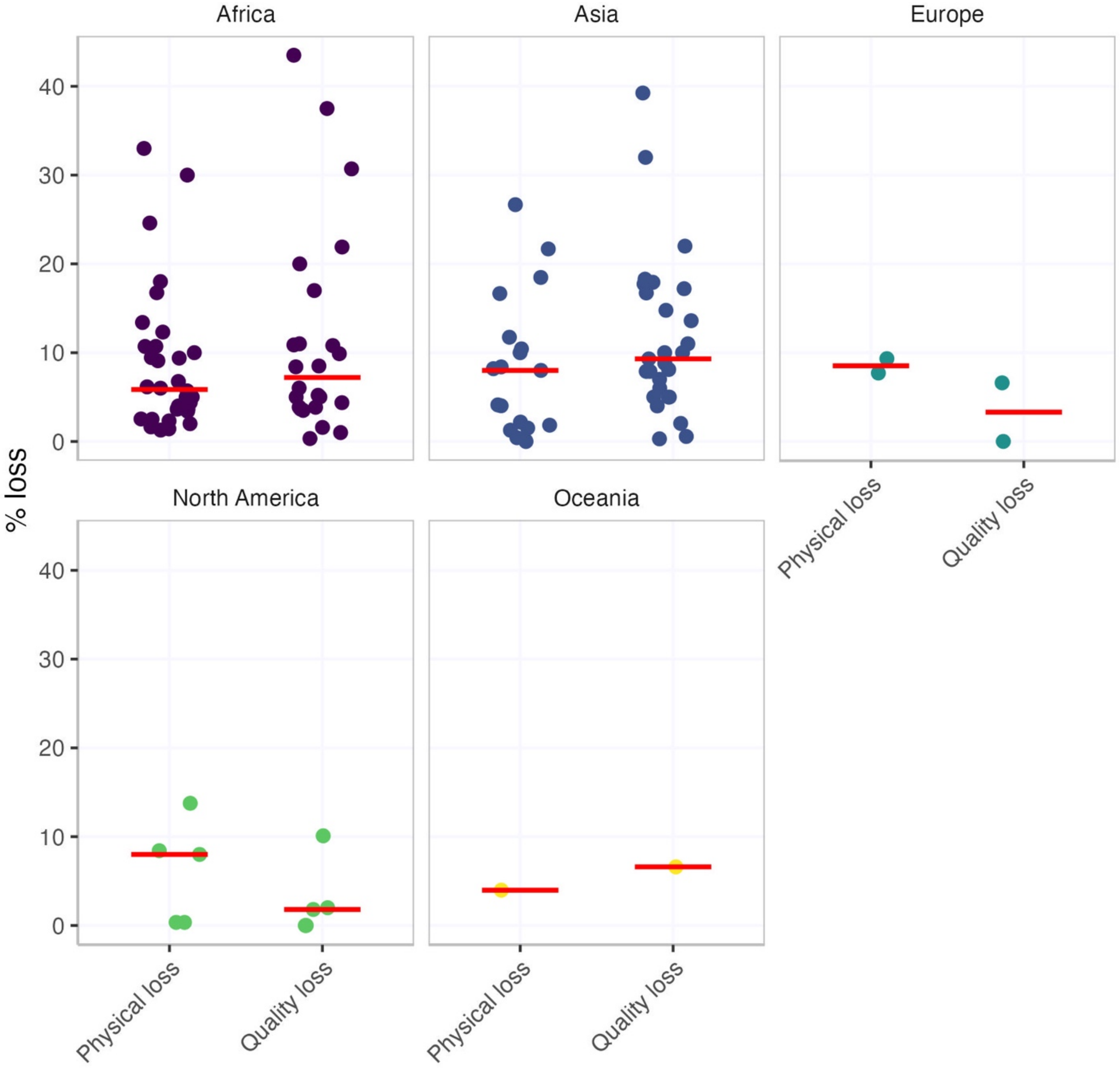

Supplementary Fig. 2. Loss of aquatic foods by region for the production and processing stages combined. Red bar at median. Values reported in Supplementary Data 3.

Estimating production method and origin of aquatic food groups

Supplementary note

We estimated the import and export quantity of each species group by production method (i.e., capture versus aquaculture) in three steps, following Love et al 2022 [1]. First, we matched each species/taxonomic group reported in production data [2] to each Harmonized System (HS) 6-digit code for aquatic food products (HS 2012 version). Each species/taxonomic group was matched based on the hierarchy of the HS code system and the description of each trade code. Species/taxonomic groups were first matched based on specific taxonomic information in the code description, with unmatched species being matched to the appropriate "not elsewhere considered" code(s). We then ensured all codes were matched to at least one species and all species were matched to at least one code. Next, we converted bilateral trade data [3] to the live weight equivalent by multiplying by the appropriate live weight conversion factor from the European Market Observatory for Fisheries and Aquaculture Products. Finally, for each country we calculated the export of each species-production method combination by multiplying the proportion of production for each species-method combination within each code by that country's exports. This results in a data set of bilateral trade flows of species/taxonomic group, with the production method information retained.

There are three important notes about these estimates are that the taxonomic resolution. First, it is limited by the resolution of the production data from the exporting country. There can be broad product categories with a mixture of product forms. For example, the bivalve category includes a mixture of shell-on and shell-off products, which can affect the product weights dramatically. Second, it does not account for preferential retention or trade routes for different species falling within the same code. Lastly, it does not account for imported products that are processed for export.

Supplementary Table 2. Weighting factors for the US aquatic food supply.

Species	% of supply [a]	% fisheries [b]	% aquaculture [b]	% imported, fisheries [b]	% imported, aquaculture [b]
Shrimp	27%	15%	85%	28%	100%
Canned tuna	14%	100%	0%	56%	0%
Salmon	16%	7%	93%	9%	99%
Alaska pollock	6%	100%	0%	0%	0%
Tilapia	8%	7%	93%	100%	93%
Catfish	3%	4%	96%	0%	0%
Pangasius	5%	0%	100%	100%	100%
Crab	3%	89%	11%	67%	100%
Cod	4%	100%	0%	25%	100%
Clams	2%	74%	26%	24%	48%
Other	11%	65%	35%	66%	84%

[a] NFI/NOAA average, 2014-2018

[b] based on US production, imports and exports for 2018. To calculate the share coming from domestic sources, subtract the share imported from 100%.

Modeling growth and biomass loss in aquaculture

Supplementary note

Growth rate and biomass loss for salmon, catfish, pangasius, and tilapia

Fish weight at a given time *t* is estimated using a von Bertalanffy growth function. In its general form this is given as:

$$W_t = W_{asa}\left(1 - e^{(-k(t-t_0))}\right)^{\alpha}$$

where W_t is the expected weight at a given time t, W_{asa} is the asymptotic average weight, t_0 is the starting point of the production process, k is the growth coefficient of the curve, α is the slope of the weight/length relationship. To represent different species, species specific parameters have been set. These are given in Supplementary Table 1.

Following previous methods [25–29], the impact of mortality can be found by determining the number of survivors as:

$$n_t = n_0.(1 - M_t(X_t))$$

where n_t is the number of survivors at time t, n_0 is the juveniles stocked at time t_0, (initial population) and $Mt(Xt)$ is the mortality rate of species X at time t. This equation is presented in exponential form:

$$n_t \cong n_0.e^{-M_t(X_t).t}$$

Specific mortality occurrences can be added with additional terms in the general function. For instance, release mortality of 5% can be added as:

$$n_t \cong n_0.e^{-M_t(X_t).t} - 0.05n_0$$

The total biomass stock of fish in a pen/pond at time *t* is then given as:

$$B_t=n_tW_t$$

The biomass loss in each period is estimated by the following equation:

$$Loss_t=n_{t1}W_t-n_tW_t$$

Mortality rate, stocking density, average cycle length, and harvested weight of salmon, catfish, pangasius, and shrimp are presented in Supplementary Data 4.

Growth rate and biomass loss for shrimp

For shrimp we used the growth function of [26]. This is given as:

$$w_t = w_i + (w_f - w_i)[(1 - k^n)/(1 - k^h)]^3$$

Where w_t is the shrimp weight after t time periods, $w_i$is the initial weight, w_f is the final weight, and *k* is the growth coefficient equal to 0.8 in this model, *h* is the time periods that have passed until harvesting time. The number of survivors, total biomass, and biomass loss for shrimp are estimated using the same equations as for the other species.

Supplementary Table 3. Parameters used to estimate growth and biomass loss at the grow-out stage.

Indicators [a]	Salmon	Catfish	Pangasius	Shrimp	Tilapia
Average mortality rate/cycle (%)	14	22	35	31	10
Stocking density (PLs)	100,000/pen	8,000/acre	200,000/acre	200/m^2	16,500/ha
Average cycle length (week)	66	30	32	21	22
Harvested weight (g/count)	4,938	663	857	30	625

[a] Parameters for species were obtained from study participants.

Supplementary Table 4. Biomass loss ratios for five species under three scenarios.

Species, Country	Time to minimum harvestable size (weeks)	Time at harvest (weeks)	Biomass loss ratio [a]		
			scenario 1	scenario 2	scenario 3
Catfish, United States	10	30	0.12	0.16	0.21
Pangasius, Vietnam	9	33	0.22	0.24	0.26
Atlantic salmon, Norway	16	81	0.06	0.08	0.11
Shrimp, Vietnam	6	21	0.12	0.17	0.22
Tilapia, China	7	22	0.06	0.09	0.14

[a] The biomass loss ratio was calculated as the total biomass at harvest divided by the sum of biomass lost weekly from the time that animals were of minimal harvestable size until the final harvest date.

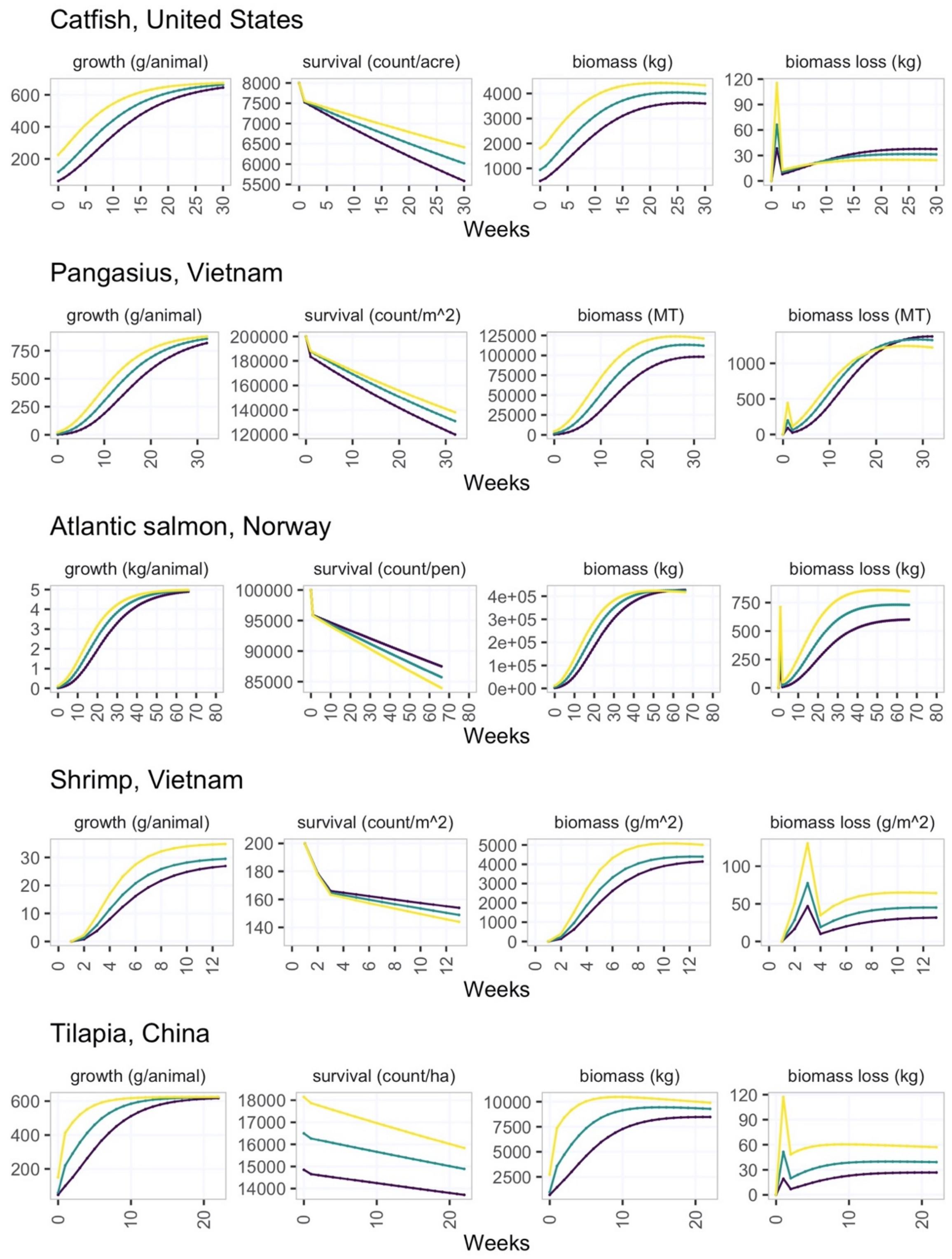

Supplementary Fig. 3. Growth, survival, biomass, and biomass loss modeled for five aquaculture species under three scenarios.

Origin of fisheries and aquaculture products and food loss

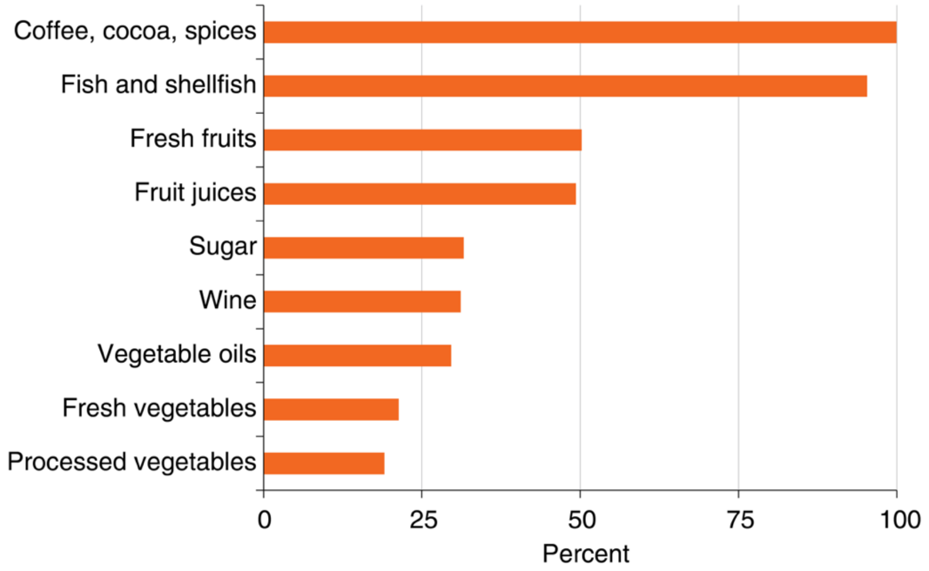

Supplementary Fig. 4 Import share of U.S. food consumption, 2011-2013. [63]

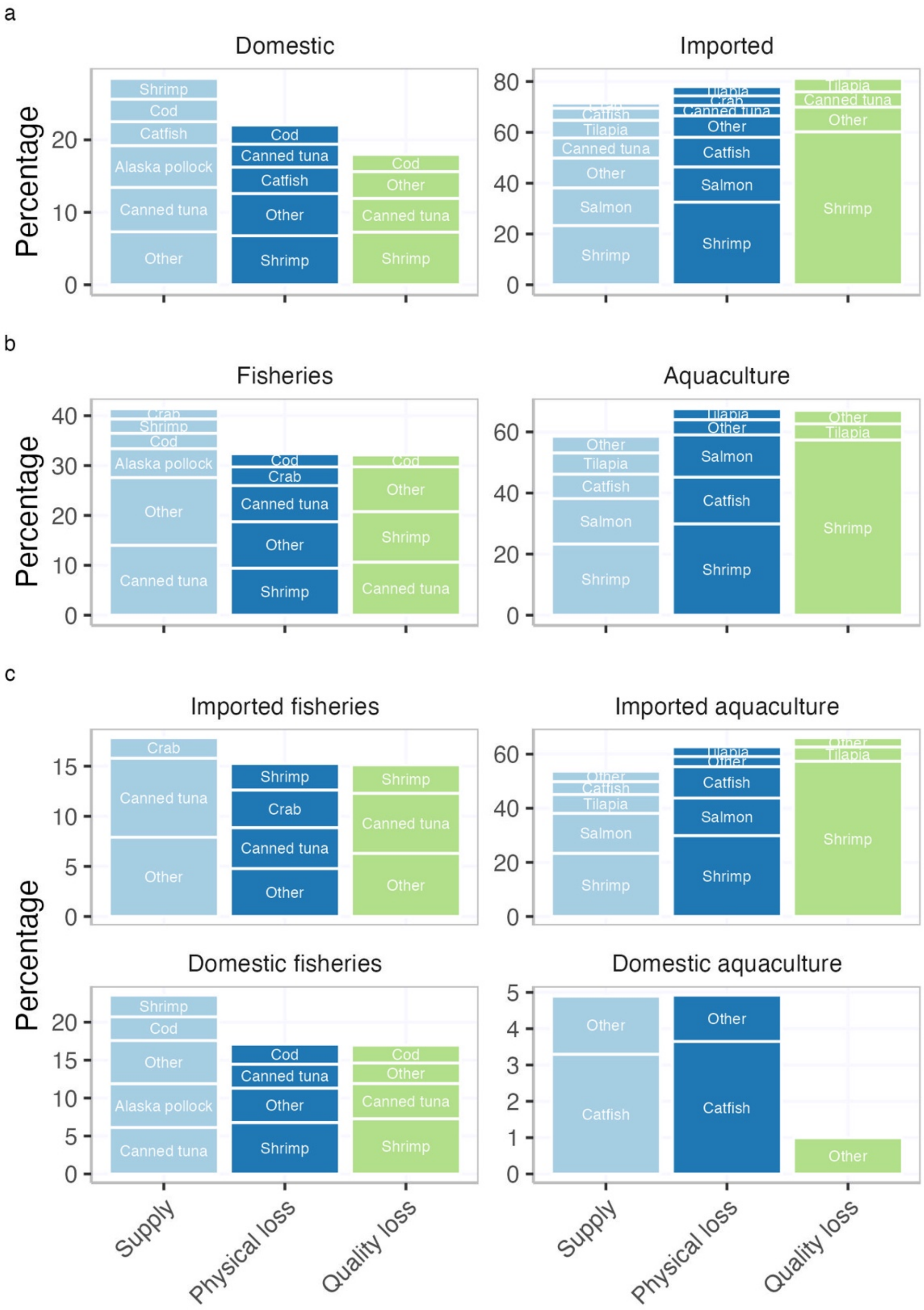

Supplementary Fig. 5. The United States aquatic food supply, physical loss, and quality loss by production method and origin. Losses < 2% of the total combined in "other" categories. (a) Origin: domestic vs imported. (b) Production method: fisheries vs aquaculture. (c) Origin and production methods combined. Within parts (a), (b), and (c); all columns of the same color sum to 100%.

Estimates of loss and waste in United States aquatic food supply chains

Supplementary Table 5. Production loss in the United States aquatic food supply.

Stage and Species group	Region	Physical loss rate	Physical loss weighted avg	Physical loss % of total	Quality loss rate	Quality loss weighted avg	Quality loss % of total	Data source
Production								
	US supply		7.54%	100%		3.39%	100%	
Fisheries								
Canned tuna	Pacific fisheries	3.90%	0.55%	7.24%	2.58%	0.36%	3.90%	[30]
Sockeye salmon	Alaska, US	0.00%	0.00%	0.00%	0.00%	0.00%	0.00%	Industry representatives Alaska US
Alaska pollock	Alaska, US	0.28%	0.02%	0.21%	0.02%	0.00%	0.28%	Industry and government representatives, Alaska, US [31,32]
Shrimp	global avg	17.86%	0.71%	9.43%	8.62%	0.34%	17.86%	[24,30]
Tilapia	global avg	4.54%	0.02%	0.33%	2.58%	0.01%	4.54%	[6,10,30,33,34]
Catfish	global avg	7.76%	0.01%	0.13%	3.95%	0.00%	7.76%	[20,35]
Pangasius	global avg	7.76%	0.00%	0.00%	3.95%	0.00%	7.76%	[20,35]
Crab	global avg	13.82%	0.42%	5.57%	2.50%	0.08%	13.82%	[22]
Cod	global avg	6.39%	0.27%	3.52%	2.58%	0.11%	6.39%	[30]
Clams	global avg	4.00%	0.06%	0.83%	0.00%	0.00%	4.00%	Industry representative, US clams
Other wild seafood	global avg	5.40%	0.39%	5.13%	2.58%	0.18%	5.40%	[30]
	Overall		5.89%			2.63%		
Aquaculture								
Shrimp	Vietnam	9.66%	2.26%	29.98%	8.33%	1.95%	9.66%	producer survey, n=5, Vietnam
Atlantic salmon	Norway	7.04%	1.05%	13.95%	0.00%	0.00%	7.04%	producer survey, n=3, Norway; [36]
Catfish	Southern US	8.34%	0.28%	3.65%	0.00%	0.00%	8.34%	producer survey, n=9, US
Pangasius	Vietnam	18.47%	0.88%	11.64%	1.03%	0.05%	18.47%	producer survey, n=7, Vietnam

Tilapia	China	3.90%	0.29%	3.87%	2.58%	0.19%	3.90%	China tilapia expert, [10,12]
Crab	global avg	6.66%	0.03%	0.34%	2.50%	0.01%	6.66%	[22]
Clams	global avg	5.76%	0.03%	0.43%	0.00%	0.00%	5.76%	Industry representative, US clams
Other farmed seafood	global avg	7.47%	0.28%	3.76%	2.58%	0.10%	7.47%	
	Overall		8.22%			3.76%		

Supplementary Table 6. Processing loss in the United States aquatic food supply.

Stage and Species group	Region	Physical loss rate	Physical loss weighted avg	Physical loss % of total	Quality loss rate	Quality loss weighted avg	Quality loss % of total	Data source
Processing								
	US supply		1.80%	100%		3.15%	100%	
Fisheries								
Canned tuna	Pacific fisheries	0.07%	0.01%	0.55%	4.03%	0.56%	17.91%	processor survey, n=1, Pacific region
Sockeye salmon	Alaska, US	0.35%	0.00%	0.20%	10.10%	0.11%	3.40%	processor survey, n=5, Alaska, US; [37]
Alaska pollock	Alaska, US	0.07%	0.00%	0.23%	1.76%	0.10%	3.22%	industry and government representatives, US, Alaska, [38]
Shrimp	global avg	3.82%	0.15%	8.44%	8.62%	0.34%	10.88%	[24,35]
Tilapia	global avg	4.55%	0.02%	1.38%	2.58%	0.01%	0.44%	[6,10–12]
Catfish	global avg	2.94%	0.00%	0.20%	3.95%	0.00%	0.15%	[20,35]
Pangasius	global avg	2.94%	0.00%	0.00%	3.95%	0.00%	0.00%	[20,35]
Crab	global avg	2.94%	0.09%	4.96%	2.50%	0.08%	2.41%	[22]
Cod	global avg	2.94%	0.12%	6.76%	4.03%	0.17%	5.30%	na
Clams	global avg	4.00%	0.06%	3.47%	0.00%	0.00%	0.00%	Industry representative, US clams
Other wild seafood	global avg	2.94%	0.21%	11.66%	4.03%	0.29%	9.15%	
	Overall		1.65%			4.03%		
Aquaculture								
Shrimp	Vietnam	2.08%	0.49%	26.99%	0.42%	0.10%	3.10%	processor survey, n=5, Vietnam; industry trade group; [39–42]
Atlantic salmon	Norway	0.66%	0.10%	5.50%	0.01%	0.00%	0.06%	processor survey, n=3, Norway; [39]
Catfish	Southern US	0.08%	0.00%	0.14%	2.00%	0.07%	2.09%	processor survey, n=3, US; [43]
Pangasius	Vietnam	0.00%	0.00%	0.00%	7.08%	0.34%	10.66%	processor survey, n=3, Vietnam; [43]
Tilapia	China	4.50%	0.34%	18.68%	11.00%	0.82%	26.08%	[10,11,20]
Crab	global avg	10.00 %	0.04%	2.15%	2.50%	0.01%	0.31%	[22]

Clams	global avg	8.00%	0.05%	2.50%	0.00%	0.00%	0.00%	Industry representative, US clams
Other farmed seafood	global avg	2.94%	0.11%	6.19%	4.03%	0.15%	4.86%	global average
	Overall		1.72%			2.28%		

Supplementary Table 7. Distribution, retail, food service and consumer loss in the United States aquatic food supply.

Stage	Description	Physical loss (%)	Quality loss (%)	Data source
Distribution	unweighted avg	1.2%	2.0%	
	returns	0.71%	1.49%	wholesale survey, n=5 (33 million kg/yr in sales)
	unsold inventory	0.45%	0.54%	“
	food safety recalls	0.06%	na	FDA food recalls [44] (1.4 million kg/yr)
Retail	weighted avg [d]	2.9%	na	retail survey (n=90) [45–47]
	fresh aquatic food	5.7%, 6.8%, 7.6%	na	“
	all frozen food [a]	0.3%, 0.8%, 1.1%	na	“
	shelf-stable food [a]	0.7%, 1.1%, 1.2%	na	“
Food service	all kitchen waste, unweighted avg [a]	9.9% [c]	na	[48–53]
Consumer	at home, weighted by income group	8.5% (6.8% - 10.6%) [b]	na	food diary survey, this study (n=70)
	away from home plate waste, unweighted avg [a,]	9.4% [c]	na	[48–60]
	Total consumer, at home and away from home weighted avg [d]	8.8%	na	

[a] not aquatic food specific values
[b] range of median values across three income groups
[c] all values reported in Supplementary Table 8
[d] weighted by data in Supplementary Table 9

Supplementary Table 8. Literature values for food service kitchen waste and plate waste.

Store type	Food type	Location	Plate waste rate (%)	Kitchen waste rate (%)	Data source
food service	all food	Switzerland	2.0%	5.8%	48
restaurant	all food	Malaysia	3.9%	6.3%	49
restaurant	all food	Malaysia	3.6%	12.1%	"
food service	all food	Malaysia	2.2%	7.3%	"
restaurant	all food	UK	8.3%	10.5%	50
food service and restaurant	all food	Sweden	8.0%	8.0%	51
restaurant	all food	Finland	9.5%	5.8%	52
café	all food	Finland	4.4%	4.4%	"
school	all food	Finland	5.7%	7.3%	"
workplace	all food	Finland	4.5%	14.2%	"
daycare	all food	Finland	4.4%	21.3%	"
food service	all food	Sweden	7.6%	15.4%	53
restaurant	all food	Canada	11.3%	na	54
university	all food	Portugal	10.5%	na	58
university	all food	Portugal	21.3%	na	59
school	entrée	US	16.5%	na	55
experimental feeding trial	meat, poultry, fish and mixtures	US	19.6%	na	56
home delivered meal	protein food	US	17.0%	na	57
middle school	entrée	US	19.2%	na	60
		Average:	9.87%	9.44%	

Supplementary Table 9. Weighting factors for aquatic food supply chains.

Stage	Value	Data source
Retail 2017-2019		
Nationally representative shares of US retail aquatic food sales		
fresh seafood	35.4%	[61] (Nielsen retail scanner data)
frozen seafood	42.0%	"
shelf-stable seafood	22.6%	"
Total	100%	
Home and away foods, 2013-2018		
Nationally representative shares of US aquatic foods consumption by food source		
At home seafood	65.7%	[62] (NHANES)
Away from home seafood	34.3%	"
total	100%	

References

1. Love, D. C. *et al.* Affordability influences nutritional quality of seafood consumption among income and race/ethnicity groups in the United States. *Am. J. Clin. Nutr.* (2022) doi:10.1093/ajcn/nqac099.
2. FishStatJ. *FAO* https://www.fao.org/fishery/en/statistics/software/fishstatj/en.
3. Gaulier, G. & Zignago, S. BACI: International Trade Database at the Product-Level (the 1994-2007 Version). (2010) doi:10.2139/ssrn.1994500.
4. Gustafsson, J., Cederberg, C. & Sonesson, U. The methodology of the FAO study: Global Food Losses and Food Waste-extent, causes and prevention”-FAO, 2011. (2013).
5. Love, D. C., Fry, J. P., Milli, M. C. & Neff, R. A. Wasted seafood in the United States: Quantifying loss from production to consumption and moving toward solutions. *Glob. Environ. Change* **35**, 116–124 (2015).
6. Akande, G. & Diei-Ouadi, Y. *Post-harvest losses in small-scale fisheries: Case studies in five sub-Saharan African countries*. (FAO, 2010).
7. Torell, E. C. *et al.* Assessing the economic impacts of post-harvest fisheries losses in Malawi. *World Development Perspectives* **19**, 100224 (2020).
8. Gyan, W. R., Alhassan, E. H., Asase, A., Akongyuure, D. N. & Qi-Hui, Y. Assessment of postharvest fish losses: The case study of Albert Bosomtwi-Sam fishing harbour, Western Region, Ghana. *Mar. Policy* **120**, 104120 (2020).
9. Diei-Ouadi, Y. *et al.* Strengthening the Performance of Post-Harvest Systems and Regional Trade in Small-Scale Fisheries: Case Study of Post-Harvest Loss Reduction in the Volta Basin Riparian Countries. Preprint at https://www.fao.org/documents/card/en/c/d9491544-87ea-47e3-a0fd-32d829b3b460/ (2015).
10. Teklu, D. Determinant Factors For Wasted Fish During Harvesting At Amerti And Fichawa Reservoirs Oromia/Ethiopia. *Journal of Fisheries Sciences* **9**, 12–15 (2015).
11. Tigabu, Y., Degebase, A. & Mohammed, A. Kinds and levels of post- harvest losses and the possible ways to reduce the losses in Lake Ziway. *ESAPproc. Ethiopian Society of Animal Production* 141–148 (2007).
12. Tigabu, Y. Fish Post Harvest Losses and Intervention Measure to Reduce Losses in Lake Hashengie. *Journal of Agricultural Development* **2**, (2012).
13. Tesfay, S. & Teferi, M. Assessment of fish post-harvest losses in Tekeze dam and Lake Hashenge fishery associations: northern Ethiopia. *Agriculture & Food Security* **6**, 1–12 (2017).
14. Kaminski, A. M. *et al.* Fish Losses for Whom? A Gendered Assessment of Post-Harvest Losses in the Barotse Floodplain Fishery, Zambia. *Sustain. Sci. Pract. Policy* **12**, 10091 (2020).

15. Ward, A. *Quantitative data on post-harvest fish losses in Tanzania. The fisheries of Lake Victoria and Mafia Island. February - 1996*. (Natural Resources Institute, 1996).

16. Kefi, A. S., Cole, S. M., Kaminski, A. M., A., W. & Mkandawire, N. L. Physical losses of fish along the value chain in Zambia: A case study of Barotse Floodplain. *International Journal of Fisheries and Aquaculture* **9**, 98–107 (2017).

17. Wibowo, S. *et al. Case studies on fish loss assessment of small-scale fisheries in Indonesia*. (FAO Fisheries and Aquaculture Circular No.1129, 2017).

18. Eyo, A. A. & Mdaihli, M. *Assessment of post-harvest losses in Nigeria fishery: the Kainji Lake model*. (FAO, 2001).

19. Mgawe, L. Y. Postharvest fish loss assessment on Lake Victoria sardine fishery in Tanzania-Rastrineobola Argentea. Preprint at (2008).

20. Nowsad, A., Hossain, M. M., Hassan, M. N., Sayem, S. M. & Polanco, J. F. Assessment of post harvest loss of wet fish: A novel approach based on sensory indicator assessment. *SAARC Journal of Agriculture* **13**, 75–89 (2015).

21. Ward, A. R. *Monsoon season post harvest losses in traditional fish processing in India*. (Natural Resources Institute, 2000).

22. Lahiri, T. *et al.* Boom and bust: Soft-shell mud crab farming in south-east coastal Bangladesh. *Aquaculture Research* vol. 52 5056–5068 Preprint at https://doi.org/10.1111/are.15377 (2021).

23. Prodhan, M. M. H., Khan, M. A., Palash, M. S. & Rahman, M. T. Nature, extent, and causes of post-harvest losses at fisher and farmer level: An in-depth study. *Aquaculture* **550**, 737856 (2022).

24. Rashid, M. M. O. & Sarkar, M. S. K. Post-harvest losses of culture, capture and marine fisheries of Bangladesh. *International Journal of Business and Economy* **2**, 11–20 (2020).

25. Thyholdt, S. B. The importance of temperature in farmed salmon growth: regional growth functions for Norwegian farmed salmon. *Aquacult. Econ. Manage.* **18**, 189–204 (2014).

26. Hernández-Llamas, A., Ruiz-Velazco, J. M. J. & Gomez-Muñoz, V. M. Economic risk associated with white spot disease and stochastic variability in economic, zootechnical and water quality parameters for intensive production ofLitopenaeus vannamei. *Rev. Aquac.* **5**, 121–131 (2013).

27. Hernandez-Llamas, A. & Ratkowsky, D. A. Growth of fishes, crustaceans and molluscs: - estimation of the von Bertalanffy, Logistic, Gompertz and Richards curves and a new growth model. *Mar. Ecol. Prog. Ser.* **282**, 237–244 (2004).

28. Yu, R. & Leung, P. Optimal Partial Harvesting Schedule for Aquaculture Operations. *Mar. Resour. Econ.* **21**, 301–315 (2006).

29. Bjørndal, T. Optimal Harvesting of Farmed Fish. *Mar. Resour. Econ.* **5**, 139–159 (1988).

30. Gilman, E. *et al.* Benchmarking global fisheries discards. *Sci. Rep.* **10**, 14017 (2020).

31. NOAA. Fisheries catch and landings reports in Alaska. *NOAA* https://www.fisheries.noaa.gov/alaska/commercial-fishing/fisheries-catch-and-landings-reports-alaska (2022).

32. Fissel, B. *et al. 2017 Stock Assessment and Fishery Evaluation Report for the Groundfish Resource of the Bering Sea/Aleutian Islands Area: Economic Status of the Groundfish Fisheries off Alasksa, 2017*. (NOAA, 2019).

33. Yared T., Abera D., Ahmed. Kinds and levels of postharvest losses and the possible ways to reduce the losses in Lake Ziway. in *Proceeding of the 15th national conferences of the Ethiopian Society of Animal Production* 141–146 (2007).

34. Yared, T. Kinds and levels of post-harvest losses and the possible ways to reduce the losses in Lake Hashengie. in *Proceeding of the sixth national conferences of the Ethiopian Fisheries and Aquatic Sciences Association* (2014).

35. Adelaja, O. A., Kamaruddin, R. B. & Chiat, L. W. Assessment of post-harvest fish losses Croaker Pseudotolithus elongatus, (Bowdich, 1825), Catfish Arius heudeloti, (Valenciennes, 1840) and Shrimp Nematopalaemon hastatus (Aurivillius, 1898) in Ondo State, Nigeria. *Aquaculture and Fisheries* **3**, 209–216 (2018).

36. Atlantic salmon and rainbow trout. *Norway Directorate of Fisheries* https://www.fiskeridir.no/English/Aquaculture/Statistics/Atlantic-salmon-and-rainbow-trout.

37. BBRSDA. BBRSDA Processor Survey. Preprint at https://static1.squarespace.com/static/56b0dfb660b5e98b87fc3d52/t/5cd5e3c6eb39313ec9967cb1/1557521372047/2018+BBRSDA+Processor+Survey+Report+Final.pdf (2019).

38. NOAA. North Pacific Groundfish Stock Assessments and Fishery Evaluation Reports. https://www.fisheries.noaa.gov/alaska/population-assessments/north-pacific-groundfish-stock-assessments-and-fishery-evaluation (2021).

39. FDA. FDA dashboards - import refusals. https://datadashboard.fda.gov/ora/cd/imprefusals.htm (2022).

40. Nirmal, N. P., Santivarangkna, C., Rajput, M. S. & Benjakul, S. Trends in shrimp processing waste utilization: An industrial prospective. *Trends Food Sci. Technol.* **103**, 20–35 (2020).

41. Ngoan, L. D., Lindberg, J. E., Ogle, B. & Thomke, S. Anatomical Proportions and Chemical and Amino Acid Composition of Common Shrimp Species in Central Vietnam. *Asian-australas. J. Anim. Sci.* **13**, 1422–1428 (2000).

42. Trung, T. S. & Phuong, P. T. D. Bioactive compounds from by-products of shrimp processing industry in Vietnam. *J. Food Drug Anal.* **20**, (2020).

43. USDA. Recalls & public health alerts. https://www.fsis.usda.gov/recalls (2022).

44. FDA. Recalls, market withdrawals, & safety alerts. *Published raw data* **2019**, (2016).

45. FMI. *The Food Retailing Industry Speaks 2015*. (Food Marketing Institute, 2015).

46. FMI. *The Food Retailing Industry Speaks 2017*. (Food Marketing Institute, 2017).

47. FMI. *The Food Retailing Industry Speaks 2019*. (Food Marketing Institute, 2019).

48. Betz, A., Buchli, J., Göbel, C. & Müller, C. Food waste in the Swiss food service industry – Magnitude and potential for reduction. *Waste Manage.* **35**, 218–226 (2015).

49. Papargyropoulou, E. *et al.* Patterns and Causes of Food Waste in the Hospitality and Food Service Sector: Food Waste Prevention Insights from Malaysia. *Sustain. Sci. Pract. Policy* **11**, 6016 (2019).

50. Collison, R. & Colwill, J. S. The analysis of food waste results and related attributes of restaurants and public houses. *J. Foodservice* **4**, 17–30 (1986).

51. Engström, R. & Carlsson-Kanyama, A. Food losses in food service institutions Examples from Sweden. *Food Policy* **29**, 203–213 (2004).

52. Silvennoinen, K., Heikkilä, L., Katajajuuri, J.-M. & Reinikainen, A. Food waste volume and origin: Case studies in the Finnish food service sector. *Waste Manag.* **46**, 140–145 (2015).

53. Eriksson, M., Persson Osowski, C., Malefors, C., Björkman, J. & Eriksson, E. Quantification of food waste in public catering services – A case study from a Swedish municipality. *Waste Manage.* **61**, 415–422 (2017).

54. von Massow, M. & McAdams, B. Table Scraps: An Evaluation of Plate Waste in Restaurants. *Journal of Foodservice Business Research* **18**, 437–453 (2015).

55. Cohen, J. F. W., Richardson, S., Bryn Austin, S., Economos, C. D. & Rimm, E. B. School Lunch Waste Among Middle School Students: Nutrients Consumed and Costs. *Am. J. Prev. Med.* **44**, 114–121 (2013).

56. Roe, B. E., Apolzan, J. W., Qi, D., Allen, H. R. & Martin, C. K. Plate waste of adults in the United States measured in free-living conditions. *PLoS One* **13**, e0191813 (2018).

57. Fogler-Levitt, E., Lau, D., Csima, A., Krondl, M. & Coleman, P. Utilization of home-delivered meals by recipients 75 years of age or older. *J. Am. Diet. Assoc.* **95**, 552–557 (1995).

58. Pinto, R. S., Pinto, R. M. D. S., Melo, F. F. S., Campos, S. S. & Cordovil, C. M.-D.-S. A simple awareness campaign to promote food waste reduction in a University canteen. *Waste Manag.* **76**, 28–38 (2018).

59. Ferreira, M., Margarida, L. M. & Rocha, A. Food waste as an index of foodservice quality. *British Food Journal* **115**, 1628–1637 (2013).

60. Smith, S. L. & Cunningham-Sabo, L. Food choice, plate waste and nutrient intake of elementary- and middle-school students participating in the US National School Lunch Program. *Public Health Nutr.* **17**, 1255–1263 (2014).

61. Love, D. C. *et al.* An Overview of Retail Sales of Seafood in the USA, 2017–2019. *Reviews in Fisheries Science & Aquaculture* **30**, 259–270 (2022).

62. Love, D. C. *et al.* Food Sources and Expenditures for Seafood in the United States. *Nutrients* **12**, (2020).

63. USDA. American diet includes many high-value imported products. https://www.ers.usda.gov/data-products/chart-gallery/gallery/chart-detail/?chartId=58398 (2016).
