# Supplementary material for: Aquatic food loss and waste rate in the United States is half of earlier estimates
Source: Nat Food. 2023 Dec 13;4(12):1058–69. doi: 10.1038/s43016-023-00881-z (PMC10727981; doi:10.1038/s43016-023-00881-z)
Supplement: Supplementary file 2 — Reporting Summary [file 43016_2023_881_MOESM2_ESM.pdf]

nature portfolio

Corresponding author(s): David Love

Last updated by author(s): Oct 13, 2023

Reporting Summary

Nature Portfolio wishes to improve the reproducibility of the work that we publish. This form provides structure for consistency and transparency in reporting. For further information on Nature Portfolio policies, see our Editorial Policies and the Editorial Policy Checklist.

Statistics

For all statistical analyses, confirm that the following items are present in the figure legend, table legend, main text, or Methods section.

n/a	Confirmed	
☐	☒	The exact sample size (n) for each experimental group/condition, given as a discrete number and unit of measurement
☒	☐	A statement on whether measurements were taken from distinct samples or whether the same sample was measured repeatedly
☒	☐	The statistical test(s) used AND whether they are one- or two-sided<br>*Only common tests should be described solely by name; describe more complex techniques in the Methods section.*
☒	☐	A description of all covariates tested
☒	☐	A description of any assumptions or corrections, such as tests of normality and adjustment for multiple comparisons
☒	☐	A full description of the statistical parameters including central tendency (e.g. means) or other basic estimates (e.g. regression coefficient) AND variation (e.g. standard deviation) or associated estimates of uncertainty (e.g. confidence intervals)
☒	☐	For null hypothesis testing, the test statistic (e.g. F, t, r) with confidence intervals, effect sizes, degrees of freedom and P value noted<br>*Give P values as exact values whenever suitable.*
☒	☐	For Bayesian analysis, information on the choice of priors and Markov chain Monte Carlo settings
☒	☐	For hierarchical and complex designs, identification of the appropriate level for tests and full reporting of outcomes
☒	☐	Estimates of effect sizes (e.g. Cohen's d, Pearson's r), indicating how they were calculated

Our web collection on statistics for biologists contains articles on many of the points above.

Software and code

Policy information about availability of computer code

Data collection	Microsoft Excel was used for data collection
Data analysis	Microsoft Excel , R v 4.2.3, and R Studio v 2023.06.1+524 were used for data analysis

For manuscripts utilizing custom algorithms or software that are central to the research but not yet described in published literature, software must be made available to editors and reviewers. We strongly encourage code deposition in a community repository (e.g. GitHub). See the Nature Portfolio guidelines for submitting code & software for further information.

Data

Policy information about availability of data

All manuscripts must include a data availability statement. This statement should provide the following information, where applicable:

- Accession codes, unique identifiers, or web links for publicly available datasets
- A description of any restrictions on data availability
- For clinical datasets or third party data, please ensure that the statement adheres to our policy

All data used to produce the results of our analysis are available in the Supplementary Information. A data availability statement is provided in the manuscript.

Research involving human participants, their data, or biological material

Policy information about studies with human participants or human data. See also policy information about sex, gender (identity/presentation), and sexual orientation and race, ethnicity and racism.

Reporting on sex and gender	Sex and gender were not considered in study design.
Reporting on race, ethnicity, or other socially relevant groupings	Race and ethnicity were not considered in study design.
Population characteristics	Population characteristics of human subjects were not collected.
Recruitment	Participants were recruited through snowball sampling and by identifying key experts through trusted networks, which may be a source of bias. To overcome this potential bias, multiple respondents per sector were interviewed to improve generalizability.
Ethics oversight	The project was approved by the Institutional Review Boards at Johns Hopkins School of Public Health (IRB# 8345) and University of Florida (IRB# 201901559).

Note that full information on the approval of the study protocol must also be provided in the manuscript.

Field-specific reporting

Please select the one below that is the best fit for your research. If you are not sure, read the appropriate sections before making your selection.

☐ Life sciences ☐ Behavioural & social sciences ☒ Ecological, evolutionary & environmental sciences

For a reference copy of the document with all sections, see nature.com/documents/nr-reporting-summary-flat.pdf

Ecological, evolutionary & environmental sciences study design

All studies must disclose on these points even when the disclosure is negative.

Study description	This study estimated aquatic food loss and waste from 2014 to 2018 for all stages of the US aquatic food supply chain.
Research sample	The sample was the US seafood supply chain, including production, processing, distribution, retail, food service and consumer stages. At the production and processing stages, we selected the top-10 species groups in the US supply for analysis and all other species groups were combined in an "other" category. In subsequent stages (distribution, retail, food service and consumption stages) we collapsed all aquatic foods together into a single category for ease of tracking product flows.
Sampling strategy	In the production and processing stages, 7 sectors were selected for primary data collection and field work. Sample sizes were intended to cover a representative number of businesses in each sector with a minimum sample size of 3 producers and 3 processors per sector. Sample sizes for primary data collection in the wholesale, retail and food service sector were limited by the number of respondents who agreed to participate. The consumer survey used a panel of U.S. participants maintained by Qualtrics.
Data collection	Data were collected from primary and secondary sources. Primary data collection was collected using interviews and surveys. Secondary data was collected from non-systematic literature reviews.
Timing and spatial scale	Data collection began in March 2019 and concluded in December 2021 . There was a break in data collection in 2020 during the pandemic.
Data exclusions	No data were excluded from the analysis.
Reproducibility	Some experimental findings were confirmed using secondary data sources or through consultation with industry experts.
Randomization	Participants were allocated into groups based on their stage of the supply chain and sector.
Blinding	Blinding was not performed for this study.

Did the study involve field work? ☒ Yes ☐ No

Field work, collection and transport

Field conditions	n/a

Location	Sampling was conducted in fisheries and aquaculture production regions of Alaska, Mississippi, Alabama, Norway and Vietnam.
Access & import/export	n/a
Disturbance	n/a

Reporting for specific materials, systems and methods

We require information from authors about some types of materials, experimental systems and methods used in many studies. Here, indicate whether each material, system or method listed is relevant to your study. If you are not sure if a list item applies to your research, read the appropriate section before selecting a response.

Materials & experimental systems

n/a	Involved in the study	
☒	☐	Antibodies
☒	☐	Eukaryotic cell lines
☒	☐	Palaeontology and archaeology
☒	☐	Animals and other organisms
☒	☐	Clinical data
☒	☐	Dual use research of concern
☒	☐	Plants

Methods

n/a	Involved in the study	
☒	☐	ChIP-seq
☒	☐	Flow cytometry
☒	☐	MRI-based neuroimaging
